# Comparative Analyses of Clearing Efficacies of Tissue Clearing Protocols by Using a Punching Assisted Clarity Analysis

**DOI:** 10.3389/fbioe.2021.784626

**Published:** 2022-01-28

**Authors:** Jiwon Woo, Eunice Yoojin Lee, Mirae Lee, Seockmo Ku, Jeong-Yoon Park, Yong Eun Cho

**Affiliations:** ^1^ Department of Neurosurgery, Graduate School of Medical Science, Brain Korea 21 Project, Yonsei University College of Medicine, Seoul, South Korea; ^2^ The Spine and Spinal Cord Institute, Department of Neurosurgery, Gangnam Severance Hospital, Yonsei University College of Medicine, Seoul, South Korea; ^3^ Biomedical Research Center, Gangnam Severance Hospital, Yonsei University College of Medicine, Seoul, South Korea; ^4^ Biomedical Research Institute, Biohedron Therapeutics Co., Ltd., Seoul, South Korea; ^5^ Columbia University Vagelos College of Physicians and Surgeons, New York, NY, United States; ^6^ Fermentation Science Program, School of Agriculture, College of Basic and Applied Sciences, Middle Tennessee State University, Murfreesboro, TN, United States

**Keywords:** transparent brains, tissue transparency, tissue clearing technique, PACA-Light, PACA-Glow

## Abstract

The advent of tissue clearing methods, in conjunction with novel high-resolution imaging techniques, has enabled the visualization of three-dimensional structures with unprecedented depth and detail. Although a variety of clearing protocols have been developed, little has been done to quantify their efficacies in a systematic, reproducible fashion. Here, we present two simple assays, Punching-Assisted Clarity Analysis (PACA)-Light and PACA-Glow, which use easily accessible spectroscopy and gel documentation systems to quantify the transparency of multiple cleared tissues simultaneously. We demonstrate the use of PACA-Light and PACA-Glow to compare twenty-eight tissue clearing protocols on rodent brains. We also show that regional differences exist in tissue transparency in the rodent brain, with cerebellar tissue consistently achieving lower clearing levels compared to the prefrontal or cerebral cortex across all protocols. This represents the largest comparative study of tissue clearing protocols to date, made possible by the high-throughput nature of our PACA platforms.

## 1 Introduction

Recent advancements in tissue clearing protocols, combined with high-resolution imaging techniques, have enabled the visualization of complex cellular networks and tissue architecture in intact, three-dimensional organs. Tissue clearing protocol is first developed as known the Spalteholtz’s method using methyl-salicylate and benzyl-benzoate to animal tissues transparent in early 1900s (Spalteholz, 1914). In the late 1980s, more modern tissue clearing methods were developed by Andrew Murray to clarify *Xenopus* eggs using benzyl-alcohol and benzyl-benzoate (BABB), also known as Murray’s clear method (Dent et al., 1989). Tissue clearing was modified the method as hydrophilic and hydrophobic protocols to applications in various tissues (Ueda et al., 2020; Vieites-Prado and Renier, 2021).

Hydrogel-based CLARITY (clear lipid-exchanged acrylamide-hybridized rigid imaging-compatible tissue-hydrogel) method (Chung et al., 2013), a number of novel techniques have been designed with the aim of improving tissue transparency and preserving structural integrity, while minimizing clearing times. These include PACT (passive clarity technique) (Yang et al., 2014), SWITCH (system-wide control of interaction time and kinetics of chemicals) (Murray et al., 2015), and MAP (magnified analysis of proteome) (Ku et al., 2016). We previously reported optimized PACT protocols and demonstrated their ability to rapidly clear the rodent brain and spinal cord (Woo et al., 2016).

Despite the surge in novel tissue clearing approaches, few methods presently exist for objective assessment and comparison of the degree of tissue transparency achieved with these protocols. Previous studies quantified the transparency of cleared brain slices using Adobe Photoshop (Lee et al., 2016); however, those methods rely on an artificial measurement of tissue transparency and do not ensure reproducibility across studies. More recent studies have begun to use spectrophotometer-based methods that measure light transmittance as a quantification of tissue transparency (d'Esposito et al., 2015; Hou et al., 2015; Chen et al., 2017; Matryba et al., 2018; Wan et al., 2018; Xu et al., 2019). Nonetheless, few offer detailed protocols that allow for standardized quantification, and no quantification methods to date can simultaneously measure the transparency of cleared tissues in a high-throughput manner.

Furthermore, studies that quantify tissue clearing do not account for regional differences; instead, they assume that processed tissues are homogenous in terms of their transparency. This fails to appreciate the high degree of heterogeneity present in all organismal tissues, which are comprised of diverse cellular networks that exist among heterogeneous extracellular matrix components. Understanding the clearing properties of intact organs in a region-specific manner can allow further optimization of tissue clearing protocols that are better suited to a particular region of interest. This, in turn, provides a deeper understanding of the three-dimensional organization of that region, which might not otherwise be uncovered.

To address these limitations, we have developed punching-assisted clarity analysis (PACA) and have introduced two novel assays for quantifying tissue clearing: PACA-Light and PACA-Glow. PACA-Light is a spectroscopy-based method that measures light absorbance and transmittance at desired light wavelengths, while PACA-Glow uses a gel documentation system to measure the amount of light permeation of cleared samples placed on a luminous disk (photoluminescent, long afterglow phosphor) (Botterman and Smet, 2015; Delgado et al., 2019; Wang et al., 2020). Both assays can be performed in 96-well plates, thereby providing a platform for high-throughput analysis of multiple cleared samples. In this study, we use PACA-Light and PACA-Glow to obtain systematic and quantitative comparisons of the efficacies of several tissue-clearing strategies, including hydrogel-based methods (Chung et al., 2013; Murray et al., 2015; Ku et al., 2016; Woo et al., 2016; Woo et al., 2020a; Woo et al., 2021), hydrophilic methods (Hama et al., 2011; Kuwajima et al., 2013; Yu et al., 2018; Zhu et al., 2019), and hydrophobic methods (Dodt et al., 2007; Erturk et al., 2012; Renier et al., 2014; Schwarz et al., 2015; Pan et al., 2016; Renier et al., 2016; Klingberg et al., 2017; Jing et al., 2018; Qi et al., 2019) in rodent brain tissues. We also reveal regional differences in tissue transparency, regardless of protocol, as cerebellar tissues consistently achieve lower degrees of clearing than the prefrontal or cerebral cortex. This work represents the largest comparative study of tissue clearing methods to date and confirms the ease and high-throughput nature of our PACA-Light and PACA-Glow assays for quantifying tissue transparency.

## 2 Materials and Methods

### 2.1 Experimental Animals

Adult male SD (Sprague Dawley) rats were purchased from Central Lab Animal Inc. (Seoul, Korea). Adult male and female ICR (Institute of Cancer Research) mice were purchased from Orient Inc. (Gyeonggi-do, Korea). Mouse embryos were isolated at E12.5 and E13.5. All animal procedures were carried out in accordance with the Guide for the Care and Use of Laboratory Animals of the Ministry of Agriculture, Food, and Rural Affairs (MAFRA), and were approved by the Institutional Animal Care and Use Committee (IACUC) at Yonsei University (license #2017-0230, Date of approval March 10, 2020; and #2015-0147, Date of approval July 1, 2016). All animal procedures were performed under veterinary supervision according to the guidelines stipulated by the Ethical Committee. Mice and rats used in these studies were cared for in accordance with the National Institutes of Health “Guide for the Care and Use of Laboratory Animals” and ARRIVE guidelines.

### 2.2 Preparation of Rodent Brain Samples for Tissue Clearing

Upon opening the thorax, an incision was made to the right atrium of the heart. Animals were perfused with equal volumes of heparin dissolved in ice-cold 0.1 M phosphate-buffered saline (PBS; Lugen Sci, Gyeonggi-do, Korea) (10 units/mL; 50 ml for rats, 20 ml for mice) and 4% paraformaldehyde (PFA; Chembio, Gyeonggi-do, Korea). The brain was isolated using methods described previously (Woo et al., 2016), cut into either 1.5 mm or 3 mm-thick slices using a knife and brain matrix (brain slicers), and placed in 4% PFA at 4°C. The sample fixation need to fixed with fresh 4% PFA, and use of inappropriate fixative (e.g., pH non-adjusted alkaline PFA) change color.

### 2.3 Punching-Assisted Clarity Analysis (PACA) Assays for Quantifying Transparency of Cleared Brain Samples

#### 2.3.1 PACA-Light

Metal eyelets (LKF Co., Ltd., Gyeonggi-do, Korea) were used to punch 3.5–6 mm discs from rodent brains for processing with each of the clearing methods described above (Supplementary Figure S1). The 3.5 mm metal eyelets were used for the small mouse brains and for rat brains cleared using organic solvent–based clearing protocols to allow for tissue shrinkage. The 6 mm metal eyelets were used for rat brains and large brains. Long plastic straws (6 mm in diameter) were used to punch discs in brain samples cleared with the Tissue-MAP method. Discs were transferred to 96-well dishes, and a spectrometer (Molecular Devices, San Jose, CA, United States) was used to measure light absorbance/transmittance at 350–850 nm. Results were analyzed with SoftMax Pro 5 software (Molecular Devices, CA, United States). Blank values were measured as the transmittance of the clearing reagents or solutions with a matching refractive index. An average of three measurements was calculated for each sample. The final transmittance (%) of each sample was normalized to the transmittance of the blank values (clearing reagents or solutions). The organic solvent–based clearing procedures use the last clearing solution for imaging to ensure refractive index matching, but these compounds interacted with the tissue culture plastic and created some blurring.

#### 2.3.2 PACA-Glow

Punched discs 3.5–6 mm in diameter from cleared brain samples were generated as described above for each of the clearing methods used in this study. Under intense illumination, 6 mm luminous disks (SrAl_2_O_4_:Eu^2+^,Dy^3+^ photoluminescent; Excitation wavelength of luminous disk: 200–450 nm, peak value: 520 nm) were exposed to light for >30 min. The discs were then placed on the luminous disks in a 96-well plate and covered with 200 μL clearing solution or refractive index matching solution according to the clearing protocol being tested. The permeation of glowing light was quantified for 30–100 s in the dark using a Gel Doc XR (Biorad Inc., CA, United States), as described previously (Woo et al., 2020b). The light penetration was compared to the light transmittance from a luminous disk in the absence of a tissue sample. An average of three measurements (mean value; unit) was calculated for each sample. Data are presented as mean ± SD (standard deviation). Whole brain slices cleared with mPACT, as well as uncleared control slices, were also placed in 10 cm dishes and subjected to the same quantification protocol.

### 2.4 Tissue Clearing Methods

Tissue clearing protocols for rodent tissue. Hydrogel-based methods as follows: psPACT (Woo et al., 2016), mPACT (Woo et al., 2016; Woo et al., 2021), SWITCH-4 (Murray et al., 2015), SWITCH-1 (Murray et al., 2015), Tissue-MAP (Ku et al., 2016). Hydrophilic tissue clearing methods as follows: Sca*l*eA2 (Hama et al., 2011), Sca*l*eS (Hama et al., 2015), *Clear* ^*T*^ (Kuwajima et al., 2013), *Clear* ^*T2*^ (Kuwajima et al., 2013), FOCM (Zhu et al., 2019), RTF (Yu et al., 2018), SeeDB (Ke et al., 2013), SeeDBp (Ke et al., 2013), SeeDB2 (Ke et al., 2016; Ke and Imai, 2018), 60% TDE (Costantini et al., 2015), 80% TDE (Costantini et al., 2015), CUBIC-L/R (Tainaka et al., 2018), CUBIC-X (Murakami et al., 2018), LUCID (Sawada et al., 2018), Ce3D (Li et al., 2017), OPTIClear (Lai et al., 2018), and MACS (Zhu et al., 2020). Hydrophobic tissue clearing methods as follows: BABB (Dodt et al., 2007), 1P-BABB (Schwarz et al., 2015), tB-BABB (Schwarz et al., 2015), 3DISCO (Erturk et al., 2012), iDISCO+ (Renier et al., 2014; Renier et al., 2016), uDISCO (Pan et al., 2016), FDISCO (Qi et al., 2019), vDISCO (Cai et al., 2019), Ethanol-ECi (Klingberg et al., 2017), PEGASOS (Jing et al., 2018).

#### 2.4.1 psPACT and mPACT

After fixation in 4% PFA, samples were incubated in 4% acrylamide (Sigma-Aldrich Inc., St. Louis, MO, United States) in 0.1 M PBS (A4P0) at 4°C for 24 h and then in 0.25% VA-044 polymerization initiator (Wako Chemicals United States, Inc., Richmond, VA, United States) in 0.1 M PBS at 4°C for 24 h. Samples were embedded in a nitrogen gas atmosphere for 5 min, and placed in a shaking incubator at 37°C for 12 h. The mPACT-A samples were transferred to A4P0 at 37°C for 6 h. Samples processed with psPACT were incubated in clearing buffer (8% sodium dodecyl sulfate (SDS; Affymetrix Inc., OH, United States) in 0.1 M PBS, pH 8.0) at 37°C until they became transparent. Samples processed with mPACT were incubated with clearing buffer supplemented with 0.5% α-thioglycerol (Sigma-Aldrich Inc., St. Louis, MO, United States) at 37°C until they became transparent. Upon achieving transparency, all samples were washed with 0.1 M PBS for 2 h and subsequently stored in *n*RIMS solution [0.8 g/ml Nycodenz (Axis-Shield Density Gradient Media, Oslo, Norway), 0.01% sodium azide (Sigma-Aldrich Inc., St. Louis, MO, United States), and 0.1% Tween-20 (Georgiachem, Suwanee, GA, United States) in 0.1 M PBS, pH 7.5].

#### 2.4.2 SWITCH

After fixation in 4% PFA, rodent brain samples were incubated in 1% or 4% glutaraldehyde (GA; Sigma-Aldrich Inc., St. Louis, MO, United States) at 4°C for 48 h. Samples were then transferred to clearing solution [100 mM SDS, 10 mM lithium hydroxide monohydrate (Duksan Pure Chemicals, Gyeonggi-do, Korea), and 40 mM boric acid (Sigma-Aldrich Inc., St. Louis, MO, United States), in 0.1 M PBS, pH 9.0] supplemented with 1% α-thioglycerol at 37°C and then at 50°C or 80°C until they became transparent. Samples were treated with fresh clearing solution every 24 h. Upon achieving transparency, the samples were washed with 0.1 M PBS for 2 h and subsequently stored in EasyIndex (PROTOS, refractive index matching solution; LifeCanvas Technilogies, Cambridge, MA, United States) for 48 h.

#### 2.4.3 Tissue-MAP

After fixation in 4% PFA, 1 mm thick mouse brain samples were washed with 0.1 M PBS at room temperature for 1 h and then incubated in 4% acrylamide and 4% PFA in 0.1 M PBS at 4°C for 30 h under light protection. The reaction was inactivated in 1% acetamide (Sigma-Aldrich Inc., St. Louis, MO, United States), 1% glycine, and 0.02% sodium azide in dH_2_O (pH 9.0) at 37°C for 12 h under light protection. Samples were subsequently incubated in Tissue-MAP solution [30% acrylamide, 0.1% bisacrylamide (Sigma-Aldrich Inc., St. Louis, MO, United States), 10% sodium acrylate (Sigma-Aldrich Inc., St. Louis, MO, United States), and 0.05% V-50 initiator (Wako Chemicals United States, Inc., Richmond, VA, United States) in 0.1 M PBS] at room temperature for 48 h under light protection, followed by embedding in Tissue-MAP solution under nitrogen gas at 45°C for 5 h. Embedded samples were immersed in denaturation solution [200 mM SDS, 200 mM sodium chloride (Sigma-Aldrich Inc., St. Louis, MO, United States), and 50 mM Tris in dH_2_O, pH 9.0] at 90°C for 72 h. Samples were then washed in 0.1 M PBS and transferred to dH_2_O for 1 day at room temperature until they expanded 4-fold in size.

#### 2.4.4 *Clear* ^*T*^ and *Clear* ^*T2*^

After fixation in 4% PFA, samples processed with *Clear* ^*T*^ were incubated in 20% formamide and 40% formamide (*vol/vol*) (Georgiachem, Suwanee, GA, United States) in 0.1 M PBS (pH 7.4) for 30 min each, followed by 80% formamide for 2 h, then 95% formamide (v/v) until they achieved optical transparency. Samples processed with *Clear* ^*T2*^ were initially incubated in a 20% formamide/10% polyethylene glycol (PEG; Georgiachem, Suwanee, GA, United States) solution for 30 min, followed by 50% formamide/20% PEG for 1 hour, and then a fresh solution of 50% formamide/20% PEG at 37°C until tissues achieved optical transparency.

#### 2.4.5 Sca*l*eA2

After fixation in 4% PFA, samples were incubated in 20% sucrose (Sigma-Aldrich Inc., St. Louis, MO, United States) in 0.1 M PBS at 4°C for 20 h. Samples were washed with 0.1 M PBS at room temperature for 5 min, then incubated in Sca*l*eA2 [4 M urea (Georgiachem, Suwanee, GA, United States), 10% glycerol (Junsei Chemical Co., Ltd., Tokyo, Japan), and 0.1% Triton X-100 (Sigma-Aldrich Inc., St. Louis, MO, United States) in 0.1 M PBS] at 4°C until tissues achieved optical transparency. Cleared samples were mounted with Sca*l*eA2 solution.

#### 2.4.6 Sca*l*eS

Sca*l*eS0 [20% (w/v) sorbitol, 5% (w/v) glycerol, 1 mM methyl-β-cyclodextrin (Sigma-Aldrich Inc., St. Louis, MO, United States), 1 mM γ-cyclodextrin (Sigma-Aldrich Inc., St. Louis, MO, United States), 1% (w/v) N-acetyl-L-hydroxyproline (Sigma-Aldrich Inc., St. Louis, MO, United States), 3% (v/v) DMSO (Sigma-Aldrich Inc., St. Louis, MO, United States) in 0.1 M PBS], Sca*l*eS1 [20% (w/v) sorbitol, 5% (w/v) glycerol, 4 M urea, and 0.2% (w/v) Triton X-100 in dH_2_O], Sca*l*eS2 [27% (w/v) sorbitol, 2.7 M urea, 0.1% (w/v) Triton X-100, and 8.3% (v/v) DMSO in dH_2_O], Sca*l*eS3 [36.4% (w/v) sorbitol, 2.7 M urea, and 9.1% (v/v) DMSO in dH_2_O], Sca*l*eS4 [40% (w/v) sorbitol, 10% (w/v) glycerol, 4 M urea, 0.2% (w/v) Triton X-100, and 20% (v/v) DMSO in dH_2_O] was prepared. After fixation in 4% PFA, samples were sequentially incubated in Sca*l*eS0, Sca*l*eS1, Sca*l*eS2, and Sca*l*eS3 at 37°C for each 3 h. Then samples were washed with 0.1 M PBS at 4°C for 30 min. After that, sample were incubated for 3 h at 37°C with Sca*l*eS4.

#### 2.4.7 CUBIC-L/R and CUBIC-X

After fixation in 4% PFA, samples of CUBIC-L/R were immersed in 50 and 100% CUBIC-L (Tokyo Chemical Industry Co., Ltd., Tyoko, Japan) at 37°C for 72 h in a shaking incubator. Samples were washed in PBS for 24 h and pre-treated in 50% CUBIC-R+ (Tokyo Chemical Industry Co., Ltd., Tokyo, Japan) at room temperature for 24 h. Samples were then incubated in 100% CUBIC-R+ solution at room temperature for 48 h. Sample of CUBIC-X were immersed in 50 and 100% CUBIC-L at 37°C for 5 days in a shaking incubator. Samples were washed in 0.1 M PBS for 24 h and treated in CUBIC-X1 (Tokyo Chemical Industry Co., Ltd., Tokyo, Japan) solution (20% imidazole in dH_2_O) at 4°C for 60 h. Samples were then incubated in CUBIC-X2 (Tokyo Chemical Industry Co., Ltd., Tokyo, Japan) solution (5% imidazole and 55% antipyrine in dH_2_O) at room temperature for 36 h.

#### 2.4.8 FOCM

After fixation in 4% PFA, samples were incubated in FOCM solution [20% urea, 30% D-sorbitol (Duksan Pure Chemicals, Gyeonggi-do, Korea), 5% glycerol in dimethyl sulfoxide (DMSO; Sigma-Aldrich Inc., St. Louis, MO, United States)] at room temperature for 72 h.

#### 2.4.9 RTF

After fixation in 4% PFA, samples were incubated in RTF-R1 solution [30% triethanolamine (Duksan Pure Chemicals, Gyeonggi-do, Korea), 40% formamide, and 30% dH_2_O] for 6 h at room temperature, followed by incubation in RTF-R2 solution (60% triethanolamine, 25% formamide, and 15% dH_2_O) for 6 h at room temperature. Samples were then immersed in RTF-R3 solution (70% triethanolamine, 15% formamide, and 15% dH_2_O) for 2 days at room temperature until they achieved optical transparency.

#### 2.4.10 SeeDB and SeeDBp

After fixation in 4% PFA, samples were embedded into boiled 1% agarose (Invitrogen, Waltham, MA, United States) in 0.1 M PBS by slowly cooling down the agarose to room temperature over 1 hour. For clearing, samples were sequentially incubated in 20, 40, and 60% D-fructose (w/v) (Duksan Pure Chemicals, Gyeonggi-do, Korea) in 0.1 M PBS for 4–8 h at 37°C. Samples were then incubated in 80% D-fructose and 100% D-fructose, each for 12 h, followed by incubation in SeeDB solution [80.2% D-fructose (w/w) in 0.1 M PBS] for 24 h at 37°C. The SeeDBp samples were then incubated in SeeDB37 solution [86.7% (w/w) D-fructose in 0.1 M PBS] for 24 h at 50°C. If performing this step at 37°C, the concentration of fructose could be increased up to 86.7%. D-fructose solutions were prepared in 0.1 M PBS. Samples were stored in SeeDB or SeeDB37 solutions at room temperature or 37°C, respectively, for up to 1 week. All fructose solutions contained 0.5% α-thioglycerol.

#### 2.4.11 SeeDB2

After fixation in 4% PFA, samples were incubated in permeabilization solution [2% (w/v) saponin (Sigma-Aldrich Inc., St. Louis, MO, United States) in 0.1 M PBS] at room temperature for 24 h. Sample were incubated in Solution-1 [1:2 mixture of Omnipaque 350 (GE healthcare, Chicago, IL, United States) and dH_2_O] with 2% (w/v) saponin at room temperature for 10 h, and then in Solution-2 (1:1 mixture of Omnipaque 350 and dH_2_O) with 2% (w/v) saponin for 10 h. The samples were incubated in SeeDB2G (Omnipaque 350 with 2% saponin) for 12 h. Cleared sample were transferred to SeeDB2G without saponin.

#### 2.4.12 Thiodiethanol-Based Clearing

After fixation in 4% PFA, samples were incubated in either 60% or 80% 2,2′-thiodiethanol (TDE; Sigma-Aldrich Inc., St. Louis, MO, United States) at 4°C until optical transparency was achieved. The solution was replaced with fresh TDE solution, and the sample was gently shaken for 24 h at room temperature until the samples became optically transparent.

#### 2.4.13 LUCID

30% sucrose [30% (w/v) sucrose in dH_2_O] was prepared for clearing solution. After fixation in 4% PFA, samples were placed in a pretreatment solution (TDE: 30% sucrose = 20:80) at 4°C for 24 h, and sample were incubated in a final solution (TDE: glycerol: 30% sucrose = 90:5:5) at 4°C for 48 h.

#### 2.4.14 OPTIClear

OPTIClear solution is an aqueous solution consisting of 20% (w/v) N-Methyl-D-glucamine (Sigma-Aldrich Inc., St. Louis, MO, United States, 66,930; as similar to N-methylglucamine), 32% (w/v) iohexol (nycodenz) and 25% (v/v) 2,2′-thiodiethanol (TDE, as similar to thiodiglycol) in dH_2_O. Fixed samples in 4% PFA were incubated in the OPTIClear solution for 6 h or more at 37°C until they achieved optical transparency.

#### 2.4.15 Ce3D

Fixed samples in 4% PFA were washed three times in 0.1 M PBS, and sample were incubated in wash buffer containing 0.2% (v/v) Triton X-100 and 0.5% (v/v) α-thioglycerol for 30–60 min at 37°C. Subsequently tissues were treated with washing buffer for 8 h at 37°C and again for 24–36 h at room temperature. The washing buffer was replaced every 10–14 h. Samples were submerged in freshly prepared Ce3D clearing solution containing 22% (v/v) N-methylacetamide (Sigma-Aldrich Inc., St. Louis, MO, United States, M26305), 0.8 g/ml iohexol (nycodenz), 0.1% (v/v) Triton X-100 and 0.5% (v/v) α-thioglycerol in 0.1 M PBS. Samples were incubated for 24 h at room temperature until they achieved optical transparency.

#### 2.4.16 MACS

The MACS protocol contains three solutions, and recipes for each solution were as follows: MACS-R0 contains 20% (v/v) m-Xylylenediamine (m-XDA; Sigma-Aldrich Inc., St. Louis, MO, United States, X1202) and 15% (w/v) D-sorbitol (Sigma-Aldrich Inc., St. Louis, MO, United States) mixed with dH_2_O, MACS-R1 was contains 40% (v/v) m-XDA and 30% (w/v) D-sorbitol dissolved in 0.1 M PBS. MACS-R2 was prepared as a mixture of 40% (v/v) m-XDA and 50% (w/v) D-sorbitol in dH_2_O. Proper heating with incubator at 37°C could promote the dissolution of D-sorbitol. Fixed samples in 4% PFA were serially incubated for 12–36 h in MACS-R0, MACS-R1, and MACS-R2 solutions.

#### 2.4.17 BABB, 1-Propanol BABB, and *Tert*-butanol BABB

Following fixation in 4% PFA, samples were embedded in 1% agarose by boiling and slowly cooling down to room temperature. Samples processed with BABB were sequentially incubated in 25, 50, 80, and 100% ethanol (Merck Millipore, Burlington, MA, United States) for 8 h each at room temperature. Samples processed with 1-propanol BABB (1P-BABB) were sequentially incubated in 25, 50, 80, and 100% 1-propanol (Merck Millipore, Burlington, MA, United States) for 8 h each at room temperature. Samples processed with *tert*-butanol BABB (tB-BABB) were sequentially incubated in 25, 50, 80, and 100% *tert*-butanol (Daejung Chemical & Metals Co. Ltd., Gyeonggi-do, Korea) for 8 h each at room temperature. After alcohol dehydration, the samples were incubated in fresh solutions of either 100% ethanol, 1-propanol, or *tert*-butanol, as appropriate, for 12 h at room temperature. Subsequently, samples were incubated in dichloromethane (DCM; Sigma-Aldrich Inc., St. Louis, MO, United States) for 1 h at room temperature. Samples were then incubated in BABB solution [1 volume of benzyl alcohol (BA; Sigma-Aldrich Inc., St. Louis, MO, United States) to 2 volumes of benzyl benzoate (BB; Duksan Pure Chemicals, Gyeonggi-do, Korea)] for 1–2 days until they achieved optical transparency.

#### 2.4.18 3DISCO

Samples fixed in 4% PFA were sequentially incubated in 50, 70, 80, and 100% tetrahydrofuran (THF; Daejung Chemical & Metals Co. Ltd., Gyeonggi-do, Korea) in dH_2_O for 2–12 h each at room temperature, followed by incubation in DCM for 2 h at room temperature, and then in dibenzyl ether (DBE; Sigma-Aldrich Inc., St. Louis, MO, United States) for 1–2 days at room temperature until they achieved optical transparency.

#### 2.4.19 iDISCO+

Samples fixed in 4% PFA were washed in 0.1 M PBS for 1 h prior to incubation in 20, 50, 80, and 100% methanol (Merck Millipore, Burlington, MA, United States) in 0.1 M PBS at room temperature for 1 h each. Samples were then washed further with 100% methanol at 4°C for 2 h, then treated with 5% hydrogen peroxide (Merck Millipore, Burlington, MA, United States) at 4°C for 12 h. Samples were subsequently rehydrated in 80, 60, 40, and 20% methanol at room temperature for 1 h each, washed with 0.1 M PBS, and then dehydrated in 20, 40, 60, 80, and 100% methanol at room temperature for 1 h each. Upon dehydration, samples were incubated in 66% DCM:33% methanol at room temperature for 3 h, followed by incubation in 100% DCM for 15 min. Samples were washed with 100% methanol before incubation in DBE for 1–2 days at room temperature until they achieved optical transparency.

#### 2.4.20 uDISCO

PFA-fixed brain samples were incubated in 30, 50, 70, 80, 90, 96, and 100% *tert*-butanol for 2–12 h at 35°C, followed by incubation in DCM for 2 h at room temperature. BABB-D4 solution were prepared by mixing BABB with diphenyl ether (DPE; Daejung Chemical & Metals Co. Ltd., Gyeonggi-do, Korea) at a ratio of 4:1 and adding 0.4% (v/v) DL-alpha-tocopherol (Vitamin E; Sigma-Aldrich Inc., St. Louis, MO, United States). Sample were then incubated in BABB-D4 solution for 1–2 days at room temperature, until they achieved optical transparency.

#### 2.4.21 FDISCO

Samples fixed in 4% PFA were incubated in 50, 70, 80, and 100% THF (pH 9.0) for 2–12 h each at 4°C, then in DBE for 1–2 days at 4°C until they achieved optical transparency.

#### 2.4.22 vDISCO

Samples fixed in 4% PFA were incubated in permilization solution [1.5% goat serum (Abcam, Waltham, MA, United States), 0.5% Triton X-100, 0.5 mM of Methyl-beta-cyclodextrin (Sigma-Aldrich Inc., St. Louis, MO, United States), 0.2% trans-1-Acetyl-4-hydroxy-L-proline (Sigma-Aldrich Inc., St. Louis, MO, United States)] and 0.05% sodium azide in 0.1 M PBS for 2 days at 37°C, and samples were washed with 0.1 M PBS at room temperature for 2 h. Sample were incubated in 50, 70, 80, and 100% THF (pH 9.0) for 2 h each at room temperature, and samples were incubated in DCM for 2 h. Samples were then incubated in BABB solution for 1–2 days at room temperature until they achieved optical transparency.

#### 2.4.23 Ethanol-ECi

Samples fixed in 4% PFA were sequentially dehydrated in 50, 70, and 100% ethanol (pH 9.0) for 5–12 h each at 4°C. The last dehydration step with 100% ethanol was performed twice. After dehydration, samples were transferred to ethyl cinnamate (ECi; Sigma-Aldrich Inc., St. Louis, MO, United States) and incubated for 1–2 days at room temperature until they achieved optical transparency.

#### 2.4.24 PEGASOS

After fixation in 4% PFA, samples were incubated in decolorization solution for 48 h at room temperature. Decolorization solution was prepared with 25% *N,N,N′,N′*-Tetrakis (2-hydroxypropyl)ethylenediamine (quadrol; Sigma-Aldrich Inc., St. Louis, MO, United States) and 5% ammonium (Duksan Pure Chemicals, Gyeonggi-do, Korea) in 0.1 M PBS. Samples were then sequentially incubated in 30, 50, and 70% *tert*-butanol in dH_2_O for 24 h, and subsequently dehydrated in tB-PEG solution [70% *tert*-butanol, 27% poly (ethylene glycol) methacrylate (Sigma-Aldrich Inc., St. Louis, MO, United States), and 3% quadrol] at room temperature for 48 h. Processed samples were immersed in BB-PEG solution [75% benzyl benzoate and 25% poly (ethylene glycol) methacrylate in 0.1 M PBS] for 48 h at room temperature until they achieved optical transparency.

### 2.5 Immunostaining and Imaging

Brain slices and punched discs from samples cleared with psPACT, SWITCH-4, or CUBIC-L/R were incubated in PBST (1% Triton X-100 in 0.1 M PBS) for 2 h and blocked with 2% bovine serum albumin (BSA; Sigma-Aldrich Inc., St. Louis, MO, United States) in PBST for 6 h. Samples were stained with anti-GFAP antibody (1:100; Abcam, Waltham, MA, United States), anti-neurofilament antibody (1:100; Biolegend Inc., CA, United States), and DAPI dye for 14–20 h. The samples were rinsed in 0.1 M PBS for 6–10 h. The samples were stained with Goat Anti-Mouse IgG H&L Alexa Fluor® 647 (Abcam, Waltham, MA, United States) and Donkey Anti-Rabbit IgG H&L Alexa Fluor® 647 (Abcam, Waltham, MA, United States) secondary antibodies conjugated to a fluorescent label in PBST. The samples for blood vessel imaging were stained with lectin dye (DyLight 594 labeled *Lycopersicon esculentum* lectin, 1:200; Vector Laboratories Inc., CA, United States) in PBST for 14–20 h. After staining, all samples were incubated in *n*RIMS for 48 h, then transferred to a confocal microscopy dish and covered with fresh *n*RIMS solution. CUBIC-L/R samples were incubated in CUBIC-R+ for 1 day, then transferred to a confocal microscopy dish and covered with CUBIC-R+ solution. All clear images of tissues were captured using an iPhone-X camera (Apple Inc., CA, United States). Confocal imaging was performed with tile scanning and z-stack using an LSM-780 microscope (Carl Zeiss, Oberkochen, Germany) at ×10 magnification [0.45 Numerical Aperture (NA), and 2.0 Working Distance (WD, mm)], and the results were processed and analyzed using the accompanying Zeiss ZEN-2 software (Carl Zeiss, Oberkochen, Germany). Three-dimensional images were edited into serial images using Imaris v8.01 software (Bitplane, Belfast, United Kingdom).

### 2.6 Statistical Analysis

Statistical analyses were performed using Microsoft Excel 2010 software (Microsoft, New Mexico, United States). Sample size and thickness were determined using a graduated ruler and patterned background OHP film (length:width = 5 mm:5 mm). All experiments quantifying sample transparency were performed in triplicate, and data are presented as the mean ± SD (standard deviation) of three separate experiments, as indicated in the text and figure legends.

### 2.7 Quantification of Fluorescence Intensity

Quantification of fluorescence intensity was performed using Zeiss ZEN-2 software. Fluorescence intensity as a function of imaging depth was determined by measuring the median signal intensity at z-stack imaging depths (rat: 1500 μm, mouse: 500 µm). The fluorescence signals of single z-stack images were measured as integrated density, using ImageJ software [National Institutes of Health (NIH), MD, United States]. The mean values of the green fluorescence signal of single z-stack images were calculated. The median fluorescence intensity (%) and integrated density (%) were normalized to the intensity of the most superficial slide.

## 3 Results

### 3.1 PACA-Light and PACA Glow: Novel Methods for Quantitative Assessment of Tissue Transparency

**FIGURE 1:**
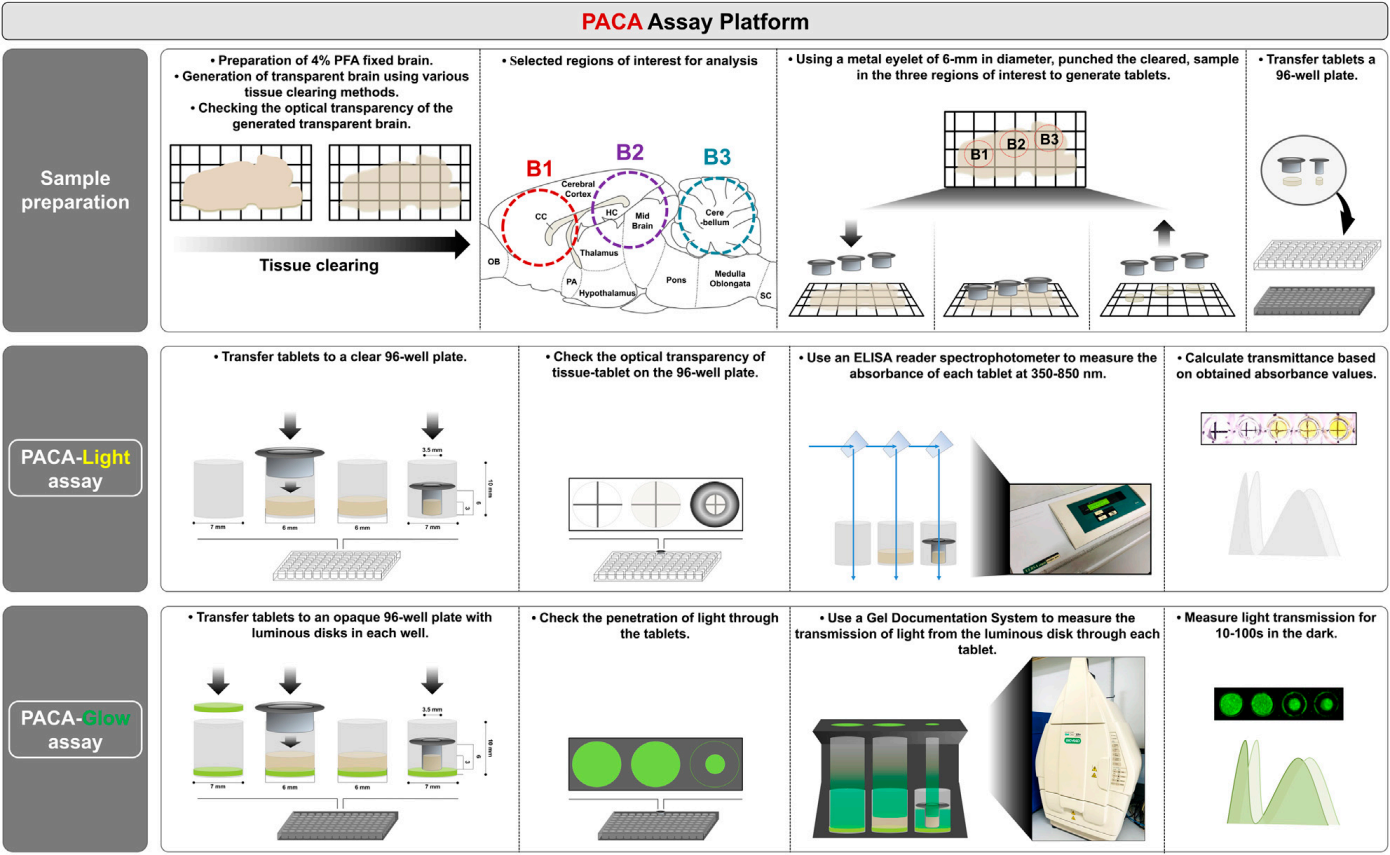
Schematization of the Punch-based Assistance Clarity-measurement Assay (PACA) platform. 1) Sample preparation for measuring tissue transparency. Cleared or uncleared samples were punched using metal eyelets, either 3.5 or 6 mm in diameter. 2) PACA-Light: Each disc was transferred to an individual well of a clear 96-well plate. An ELISA reader spectrophotometer was used to measure light absorbance along a range of wavelengths. Transmittance was analyzed based on the obtained absorbance values. 3) PACA-Glow: Each disc was transferred to an individual well of a black, opaque 96-well plate with a luminous disk (SrAl_2_O_4_:Eu^2+^, Dy^3+^ photoluminescent). A gel documentation system was used to analyze the transmission of light from the luminous disk through the disc. Details of the process applied are provided in Supplementary Figure S1.

While a variety of tissue clearing methods have been established since the development of CLARITY (Chung et al., 2013), manual methods that can accurately and precisely quantify the transparency of cleared tissues are still lacking. We developed a novel spectrophotometer-based assay, PACA-Light, which allows for efficient and cost-effective high-throughput analysis of cleared samples. PACA-Light can compare and analyze tissue clearing efficiency for different methods based on transmittance, as well as sample size changes through the use of different sizes of metal eyelets. As depicted in Figure 1 (see also Supplementary Figure S1), PACA-Light uses 3.5–6 mm metal eyelets to punch round discs from a cleared tissue sample. In our studies, we used 1.5–3 mm-thick cleared sagittal sections of rodent brains, and generated three round discs, each from a different region of the brain: the prefrontal cortex and basal ganglia (B1), cerebral cortex and midbrain/diencephalon (B2), and cerebellum (B3). The absorbance and transmittance were measured for each sample’s secondary antibodies with a spectrophotometer at a range of 350–850 nm. Results are compared to control wells that are either blank or filled with the final solution used to generate the cleared samples.

**FIGURE 2:**
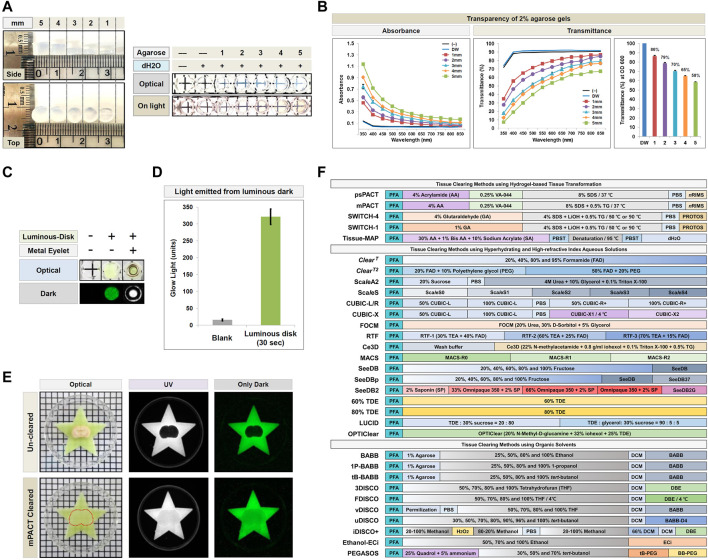
Demonstration of the PACA platform and schematic of the tissue clearing methods used in the present study. **(A,B)** Absorbance and transmittance (%) of 2% agarose gels of varying thickness (1–5 mm) at 350–850 nm. Each color line and bar point to assessment values of dH_2_O (DW; blue) and 2% agarose gel discs of differing thickness. Red (square), 1 mm; Violet (round), 2 mm; sky blue (triangle), 3 mm; Yellow (diamond), 4 mm; Green (square), 5 mm. All experiments quantifying gel transparency were performed in triplicate, and data are presented as the mean ± SD (standard deviation) of three separate experiments. **(C)** Comparison of light emitted by the luminous disk. **(D)** Quantification of light transmitted from the luminous disk (SrAl_2_O_4_:Eu^2+^, Dy^3+^ photoluminescent; Excitation wavelength of luminous disk: 200–450 nm, peak value: 520 nm) exposure for 30 s in the dark, and compared to a blank control. An average of three measurements (glow light mean value: unit) was calculated for the luminous disk using Gel Doc XR. **(E)** Comparison of light transmission between an uncleared 4 mm thick mouse brain and a sample cleared with mPACT on a luminous disk in both UV and dark conditions. The transparency and size of the sample was evident against a patterned background (length:width = 5 mm:5 mm). **(F)** Schematic representation of the 32 protocols of the 28 tissue clearing techniques used in this comparative study.

We first performed proof-of-concept analyses with discs generated from 2% agarose gels, 1–5 mm in thickness, in distilled water (dH_2_O) for a matching refractive index (Figures 2A,B). For both absorbance and transmittance, as the wavelength increased, we observed a decrease in absorbance and an increase in transmittance. Furthermore, as expected, for every 1 mm increase in gel thickness, we observed an increase in absorbance and a decrease in transmittance at each wavelength. These results demonstrated the potential for applying this technique to quantify the resulting transparency of tissues processed with CLARITY-based methods.

In addition to PACA-Light, we developed a second PACA assay, PACA-Glow, which involves placing cleared samples over a luminescent disk (SrAl_2_O_4_: Eu^2+^, Dy^3+^ photoluminescent) and quantifying the amount of light transmitted through the sample. Luminescent disks measuring 6 mm in diameter were inserted into individual wells of an opaque 96-well plate, and the amount of light transmitted from these disks was quantified in the dark using a gel documentation system (Figure 2C). While wells without luminescent disks showed little to no light transmittance, wells with luminescent disks exposed to light for >30 min demonstrated approximately 320 units of transmitted light (Figure 2D, Supplementary Figure S2). As a proof-of-concept, we imaged light permeation through brain slices cleared with mPACT, as well as uncleared brain samples as a control. As shown in Figure 2E, while no light permeated through uncleared brains, a significant amount of light permeated the mPACT-cleared brains.

### 3.2 Application of PACA-Light and PACA-Glow to Assess Tissue Transparency Achieved With Hydrogel-Based Tissue Clearing Protocols

Upon demonstrating the feasibility and efficiency of the PACA-Light and PACA-Glow assays, we sought to apply these techniques for quantitative comparisons of the efficacies of currently established tissue clearing techniques. As listed in Figure 2F, these include CLARITY-based and hydrogel-based tissue clearing methods (psPACT, mPACT, SWITCH-1, SWITCH-4, and Tissue-MAP), hydrophilic tissue clearing methods (*Clear* ^*T*^, *Clear* ^*T2*^, Sca*l*eA2, Sca*l*eS CUBIC-L/R, CUBIC-X, Ce3D, MACS, FOCM, RTF, SeeDB, SeeDBp, SeeDB2, 60% TDE, 80% TDE, LUCID, and OPTIClear), hydrophobic tissue clearing methods that employ organic solvents (ethanol-BABB, 1-propanol BABB, *tert*-butanol BABB, 3DISCO, FDISCO, vDISCO, uDISCO, iDISCO+, Ethanol-ECi and PEGASOS). To date, no single study has compared the clearing efficacies of CLARITY-based methods at this scale in a high-throughput, quantitative manner.

**FIGURE 3:**
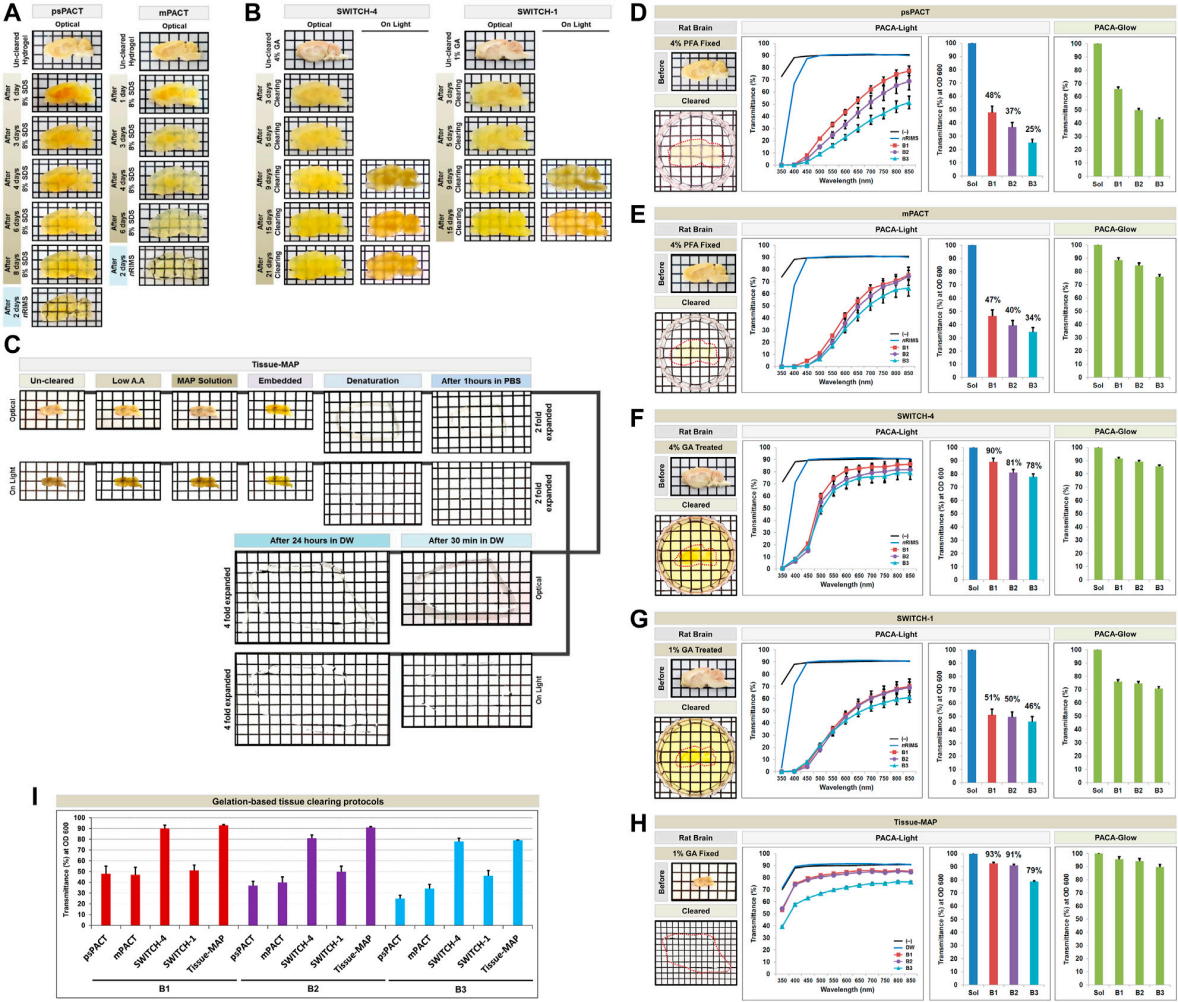
Comparison of tissue clearing achieved with hydrogel-based tissue clearing protocols. Comparison of the optical clearing processes of PACTs (psPACT and mPACT) **(A)**, SWITCHs (SWITCH-1 and SWITCH-4) **(B)**, and Tissue-MAP **(C)** in rat brain samples (3 mm thickness). Comparison of clearing efficacies of psPACT **(D)**, mPACT **(E)**, SWITCH-4 **(F)**, SWITCH-1 **(G)**, and Tissue-MAP **(H)** in rodent brain samples with both PACA-Light and PACA-Glow. Optical images showing samples after clearing (red dotted line), along with any changes in sample size upon tissue processing, are included (also Supplementary Figure S12). **(I)** Comparison of clearing efficacies of hydrogel-based tissue clearing protocols. Three discs from three distinct brain regions (B1: prefrontal cortex and basal ganglia, B2: cerebral cortex and midbrain/diencephalon, B3: cerebellum) were generated and analyzed for each sample. Each color line and bar point to assessment values of empty (black) and refractive index matching solution (blue) of each protocol, and three distinct regions of the brain the B1 (square, red), B2 (diamond, violet), and B3 (triangle, sky blue). Green bars point to assessment values of glow light (unit) in dark. Results reflect three replicates of each experiment, and data are presented as the mean ± SD (standard deviation). The transparency of the cleared brain was evident against a patterned background (length:width = 5 mm:5 mm).

We first quantified the transparency of rat and mouse brains cleared with five gelation-based protocols. Brain slices (3 mm thick for rats and 1.5 mm thick for mice) were used for psPACT, mPACT, SWITCH-1, and SWITCH-4. The psPACT and mPACT protocols resulted in tissue clearing in 2–8 days, while SWITCH-1 and SWITCH-4 achieved clearing in 2–25 days. For Tissue-MAP, we used 1 mm thick mouse brain slices, which expanded four-fold in size and thickness during the 3 days required to achieve optical transparency (Figures 3A–C). The Tissue-MAP sample showed limited transparency <6 mm thick in the PACA platform, because of its characteristic four-fold expansion of the tissue-hydrogel hybrid, which also limited its use in 96-well plates. For each sample, we evaluated PACA-Light and PACA-Glow by generating 6 mm discs from three brain regions: prefrontal cortex and basal ganglia (B1), cerebral cortex and midbrain/diencephalon (B2), and cerebellum (B3) (Figures 3D–H, Supplementary Figure S3). Empty wells and wells filled with the final clearing solution for each protocol were used as controls. We observed significant differences in transparency between brain regions, as well as across protocols for the same region. Overall, PACA-Light demonstrated higher levels of light transmittance for SWITCH-4 and Tissue-MAP for all three brain regions (Figures 3F,H). B2 and B3 showed decreased light transmittance relative to B1 across all protocols, and this effect was more pronounced in samples treated with psPACT and mPACT (see also Figures 3D,E). Results obtained with PACA-Glow were consistent with the trends observed with PACA-Light, although the differences in clearing between brain regions treated with psPACT and mPACT were more pronounced when analyzed with PACA-Light (Figure 3I). Similar results were obtained for mouse brain samples processed with the same protocols (Supplementary Figure S4). We also used mPACT to generate clear non-CNS tissues, such as kidney, lung, gland, heart, stoma, and spleen, and quantified their transparencies with PACA-Light (Supplementary Figure S5).

### 3.3 Comparison of Tissue Transparency Achieved With Protocols Using Hydrophilic Tissue Clearing Protocols

**FIGURE 4:**
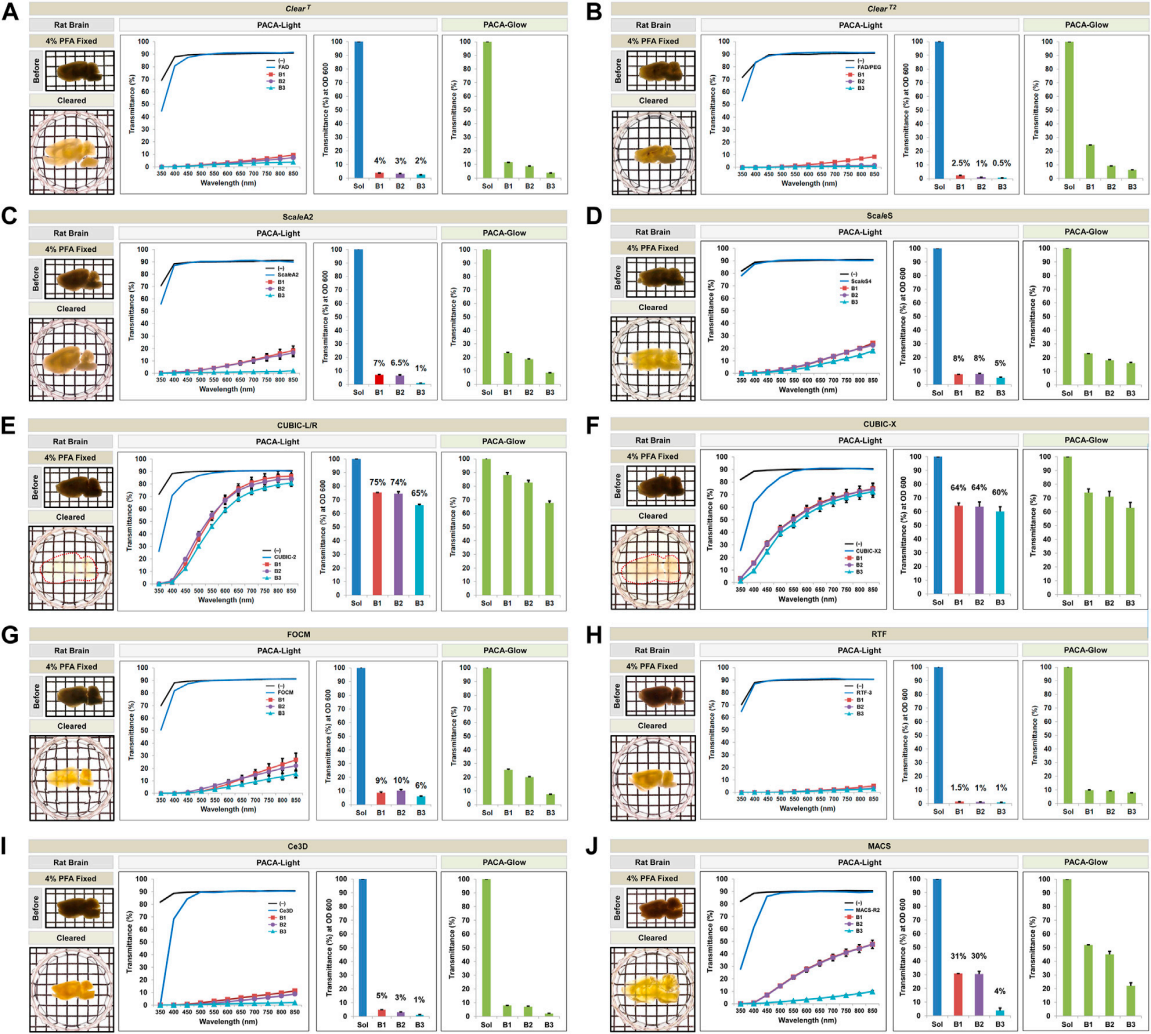
Comparison of tissue clearing achieved with ten hydrophilic tissue clearing methods. Comparison of clearing efficacies of *Clear* ^*T*^ **(A)**, *Clear* ^*T2*^ **(B)**, Sca*l*eA2 **(C)**, Sca*l*eS **(D)**, CUBIC-L/R **(E)**, CUBIC-X **(F)**, FOCM **(G)**, RTF **(H)**, Ce3D **(I)**, and MACS **(J)** on rat brain samples (3 mm thickness) with both PACA-Light and PACA-Glow. All tissue clearing time course are shown in Supplementary Figures S6, S7. Optical images showing samples after clearing (red dotted line), along with any changes in sample size upon tissue processing, are included (also Supplementary Figure S12). Three discs from three distinct brain regions (B1: prefrontal cortex and basal ganglia, B2: cerebral cortex and midbrain/diencephalon, B3: cerebellum) were generated and analyzed for each sample. Each color line and bar point to assessment values of empty (black) and refractive index matching solution (blue) of each protocol, and three distinct regions of the brain the B1 (square, red), B2 (diamond, violet), and B3 (triangle, sky blue). Green bars point to assessment values of the glow light (unit) in the dark. Results represent three replicates of each experiment, and data are presented as the mean ± SD (standard deviation). The transparency of the cleared brain was evident against a patterned background (length:width = 5 mm:5 mm).

**FIGURE 5:**
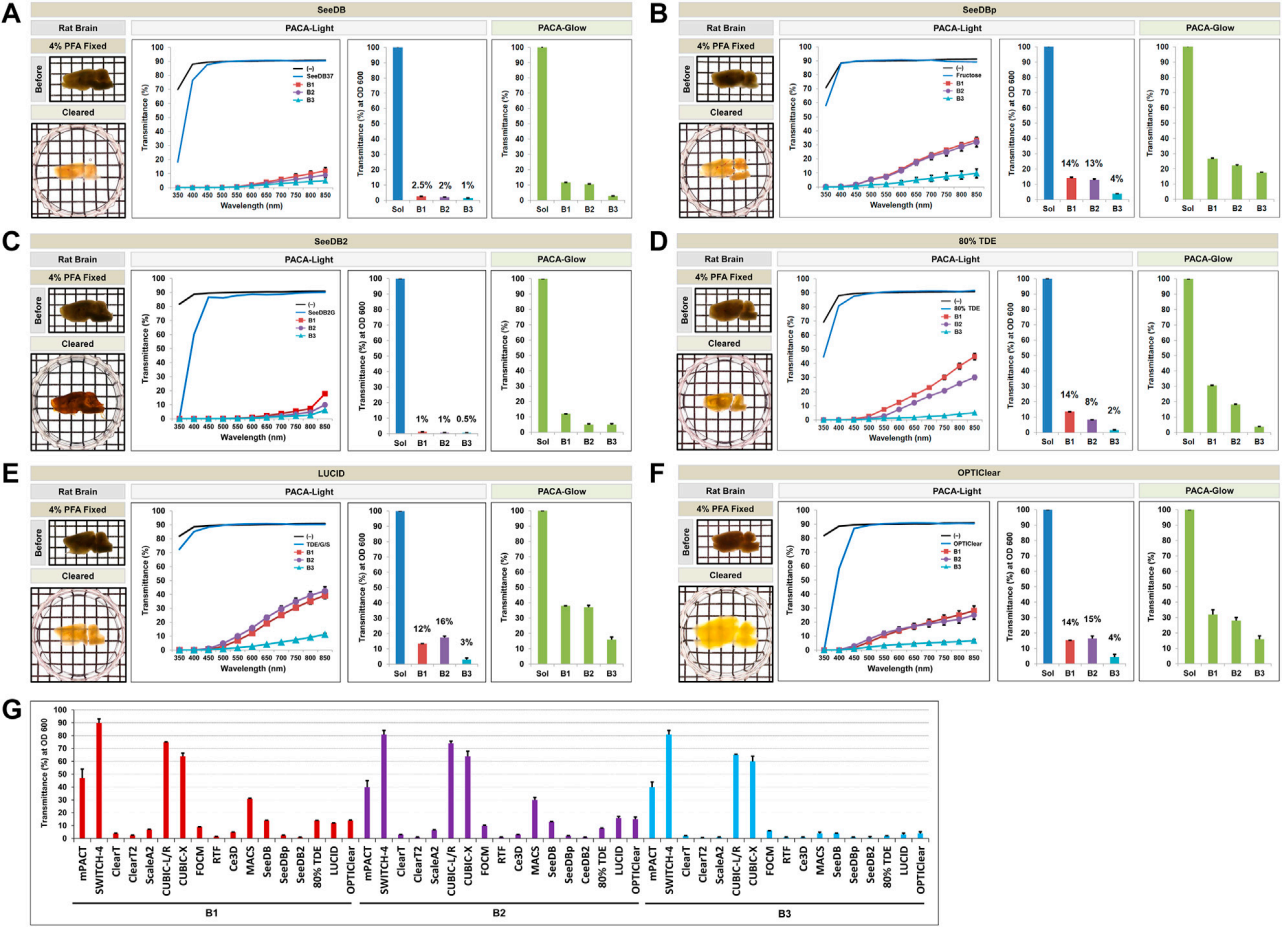
Comparison of tissue clearing achieved with six hydrophilic tissue clearing methods. Comparison of clearing efficacies of SeeDB **(A)**, SeeDBp **(B)**, SeeDB2 **(C)**, 80% TDE **(D)**, LUCID **(E)**, and OPTIClear **(F)** on rat brain samples (3 mm thickness) with both PACA-Light and PACA-Glow. All tissue clearing time course are shown in Supplementary Figures S6, S7. Optical images showing samples after clearing (red dotted line), along with any changes in sample size upon tissue processing, are included (see also Supplementary Figure S12). **(G)** Comparison of clearing efficacies of sixteen hydrophilic tissue clearing. Three discs from three distinct brain regions (B1: prefrontal cortex and basal ganglia, B2: cerebral cortex and midbrain/diencephalon, B3: cerebellum) were generated and analyzed for each sample. Each color line and bar point to assessment values of empty (black) and refractive index matching solution (blue) of each protocol, and three distinct regions of the brain the B1 (square, red), B2 (diamond, violet), and B3 (triangle, sky blue). Green bars point to assessment values of the glow light (unit) in the dark. Results represent three replicates of each experiment, and data are presented as the mean ± SD (standard deviation). The transparency of the cleared brain was evident against a patterned background (length:width = 5 mm:5 mm).

We also applied the same PACA-Light and PACA-Glow assays to 1.5 mm mouse or 3 mm thick rat and brain slices processed using hyperhydrating solutions or aqueous solutions with high refractive indices (Supplementary Figures S6, S7). Samples cleared with *Clear* ^*T*^ and *Clear* ^*T2*^ achieved clearing 24 h after incubation with 95% formamide (FAD) and 50% FAD/20% polyethylene glycol, with minimal fluctuation in size after clearing. FOCM, RTF, SeeDB, SeeDBp, and SeeDB2 also caused little to no size fluctuation after clearing, and tissue clearing was achieved in varying timeframes, from 48 h to 1 week. Rat brain samples processed with Sca*l*eA2, Sca*l*eS, CUBIC-L/R, CUBIX-X, and OPTIClear expanded in size and thickness after clearing, and brains cleared using 80% TDE shrank relative to their original size (Figures 4, 5, and Supplementary Figure S12). Similar results were obtained for mouse brain samples processed with the same protocols (Supplementary Figure S8).

Qualitatively, CUBIC-L/R and CUBIC-X achieved the best clearing observed by eye. This was consistent with quantitative results obtained with PACA-Light and PACA-Glow, which showed minimal clearing by all other hydrophilic tissue clearing techniques except two CUBIC protocols (CUBIC-L/R and CUBIC-X), which showed transmittance values from 60 to 75% in both PACA-Light and PACA-Glow assays (Figures 4E,F). Consistent with the trends observed in samples cleared using gelation-based methods, B3 (cerebellum) demonstrated the lowest amount of light transmittance in both assays, regardless of which hydrophilic protocol was used (Figure 5G).

### 3.4 Comparison of Tissue Transparency Achieved With Hydrophobic Tissue Clearing Protocols

**FIGURE 6:**
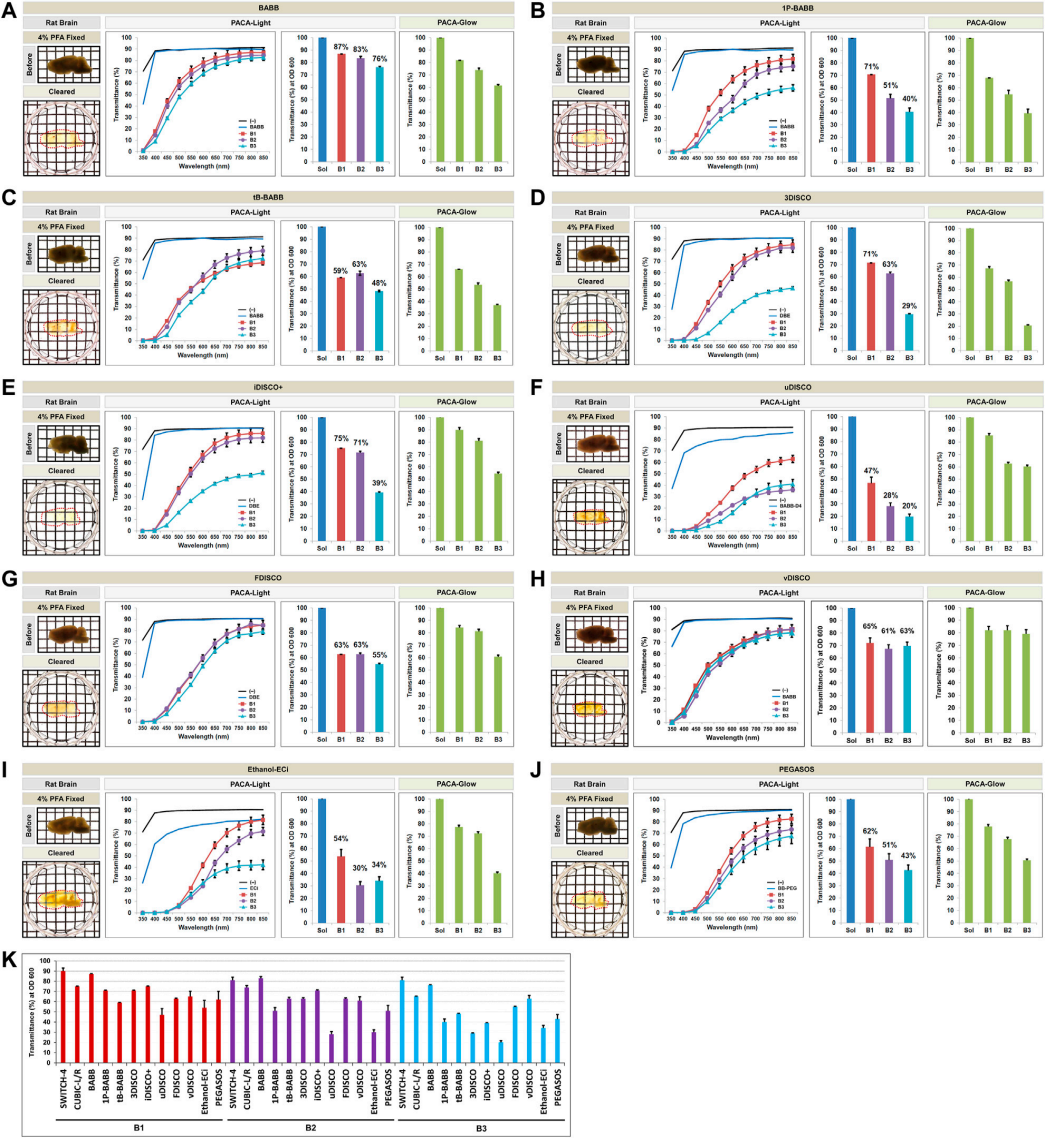
Comparison of tissue clearing achieved in rat brains processed with hydrophobic tissue clearing protocols. Comparison of clearing efficacies of Ethanol BABB **(A)**, 1-propanol BABB **(B)**, *tert*-butanol BABB **(C)**, 3DISCO **(D)**, iDISCO+ **(E)**, uDISCO **(F)**, FDISCO **(G)**, vDISCO **(H)**, Ethanol-ECi **(I)**, and PEGASOS **(J)** in rat brain samples (3 mm thickness) with both PACA-Light and PACA-Glow. All tissue clearing time course are shown in Supplementary Figure S9. Optical images show samples after clearing (red dotted line), as well as any changes in sample size upon tissue processing (also Supplementary Figure S12). **(K)** Comparison of clearing efficacies of ten hydrophobic tissue clearing protocols. Three discs from three distinct brain regions (B1: prefrontal cortex and basal ganglia, B2: cerebral cortex and midbrain/diencephalon, B3: cerebellum) were generated and analyzed for each sample. Each color line and bar point to assessment values of empty (black) and refractive index matching solution (blue) of each protocol, and three distinct regions of the brain the B1 (square, red), B2 (diamond, violet), and B3 (triangle, sky blue). Green bars point to assessment values of glow light (unit) in dark. Results represent three replicates of each experiment, and data are presented as the mean ± SD (standard deviation). The transparency of the cleared brain was evident against a patterned background (length:width = 5 mm:5 mm).

We also compared hydrophobic tissue clearing protocols that use organic solvents (Supplementary Figure S9), as outlined in Figure 2F. The organic solvent–based clearing procedures used the last clearing solution for imaging due to refractive index matching, and these compounds interacted with the tissue culture plastic of the 96-well plate and created blurring. Therefore, in the PACA-Light analysis, the transmittance (%) of each sample was normalized to the transmittance of the last clearing solution (blank values) with PACA-Light. We first tested variations of the BABB protocol; the original ethanol-based BABB method involves serial dehydration in 50, 80, and 100% ethanol after embedding in 1% agarose and achieved tissue clearing 24 h after incubation in the final BABB solution, with slight shrinkage in sample size (Figure 6A). Modified BABB protocols that used either 1-propanol or *tert*-butanol instead of ethanol also achieved tissue clearing 24 h after incubation in BABB, though to a lesser degree and with greater shrinkage (Figures 6B,C). Therefore, the samples cleared with organic solvents were qualitatively evaluated after punching using a 3.5 mm metal eyelet. Qualitative observations were consistent with quantitative measurements of tissue clearing obtained with PACA-Light and PACA-Glow, which showed higher levels of light transmittance for samples processed with ethanol BABB than with 1-propanol BABB and *tert*-butanol BABB (Figures 6A–C).

We also tested the original 3DISCO method and its variations, iDISCO+, uDISCO, FDISCO and vDISCO. The 3DISCO process, which employs serial dehydration and delipidation in gradients of tetrahydrofuran (THF), achieved tissue clearing after 24 h in the final dibenzyl ether (DBE) solution, with moderate tissue shrinkage (Figure 6D). The uDISCO, FDISCO and vDISCO protocols also resulted in tissue shrinkage, without noticeable improvements in tissue transparency (Figures 6F–H). The iDISCO + method, which employs serial dehydration in methanol gradients, achieved comparable levels of tissue transparency while preserving both tissue size and thickness (Figure 6E). Quantitative assessment using PACA-Light and PACA-Glow was consistent with these observations, as 3DISCO and iDISCO + samples demonstrated similar levels of light transmittance for all three brain regions. Those values were lower for uDISCO and FDISCO (Figures 6D–H). The PEGASOS ethanol-based method showed higher levels of light transmittance for samples processed with Ethanol-ECi (Figures 6I,J). Consistent with our previous observations, regardless of the protocol used, B3 (cerebellum) samples consistently showed the lowest levels of light transmittance, while B1 (prefrontal cortex) showed the highest levels (Figure 6K). These results were also similar in mouse brain samples processed with the same protocols (Supplementary Figures S10, S11). The same held true for tissue discs from brain slices of different thickness. We observed a difference in transmittance with sample thickness in three distinct regions of the mouse hemibrain at each wavelength (Supplementary Figures S13, S14). These results indicate that thicker slices of transparent brain will have less transmittance. The PACA-Light and PACA-Glow transmittance values obtained for all clearing protocols and tissues tested in this study are listed in Table 1 (also Supplementary Table S1 for mouse brain).

**TABLE 1:** Comparison of tissue clearance achieved in rat brains processed *via* various tissue clearing methods in this study.

Tissue clearing method	3-mm thick rat brain slice								
	PACA-light						PACA-glow		
	Minimal transmittance (%)						Transmittance (%)		
	OD 600			OD 850			30-100 s		
	B1	B2	B3	B1	B2	B3	B1	B2	B3
psPACT	±48	±37	±25	±86	±77	±57	±66	±50	±43
mPACT	±47	±40	±34	±83	±83	±72	±89	±85	±76
SWITCH-1	±51	±50	±46	±78	±76	±67	±76	±75	±70
SWITCH-4	±90	±81	±78	±95	±90	±87	±92	±89	±86
*Clear^T^*	±4	±3	±2	±10	±8	±4	±12	±9	±4
*Clear^T2^*	±2.5	±1	±0.5	±9	±2	±1	±25	±9	±6
Sca*l*eA2	±7	±6.5	±1	±20	±18	±2	±52	±41	±19
Sca*l*eS	±8	±8	±5	±27	±25	±20	±23	±18	±16
CUBIC-L/R	±75	±74	±65	±96	±93	±89	±88	±83	±68
CUBIC-X	±64	±64	±60	±83	±82	±80	±74	±71	±63
FOCM	±9	±10	±6	±30	±24	±17	±25	±20	±8
RTF	±1.5	±1	±1	±6	±4	±3	±10	±9	±8
Ce3D	±5	±3	±1	±13	±10	±2	±8	±7	±2
MACS	±31	±30	±4	±53	±53	±11	±52	±45	±22
SeeDBp	±14	±13	±4	±38	±36	±11	±27	±22	±17
SeeDB	±2.5	±2	±1	±13	±10	±5	±12	±10	±3
SeeDB2	±1	±1	±0.5	20	±11	±7	±12	±5	±5
80% TDE	±14	±8	±2	±49	±33	±6	±30	±18	±4
LUCID	±13	±17	±3	±44	±47	±12	±38	±37	±16
OPTIClear	±15	±16	±4	±31	±28	±7	±32	±28	±16
BABB	±87	±83	±76	±97	±94	±92	±82	±74	±61
1P-BABB	±71	±51	±40	±91	±84	±63	±68	±55	±40
tB-BABB	±59	±63	±48	±77	±88	±81	±66	±53	±37
3DISCO	±71	±63	±29	±94	±91	±51	±67	±57	±21
iDISCO+	±75	±71	±39	±95	±91	±60	±90	±81	±55
uDISCO	±47	±28	±20	±73	±42	±48	±85	±63	±60
FDISCO	±63	±63	±55	±94	±94	±88	±84	±81	±61
vDISCO	±72	±67	±70	±90	±90	±87	±82	±82	±79
Ethanol-ECi	±54	±30	±34	±99	±87	±51	±77	±72	±40
PEGASOS	±62	±51	±43	±92	±81	±85	±78	±68	±51

**FIGURE 7:**
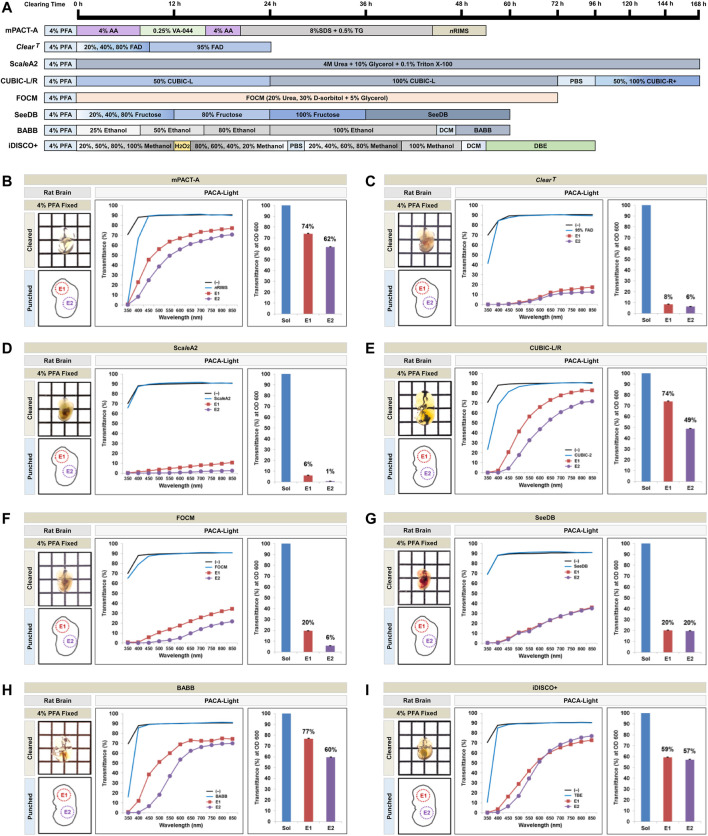
Comparison of tissue clearing achieved in mouse embryos processed with various clearing protocols. **(A)** Timeline of eight passive tissue clearing methods in mouse embryo. The individual reagents or processes used for dehydration and clearing process in the clearing methods are shown. Comparison of clearing efficacies of mPACT-A **(B)**, *Clear* ^*T*^ **(C)**, Sca*l*eA2 **(D)**, CUBIC-L/R **(E)**, FOCM **(F)**, SeeDB **(G)**, Ethanol BABB **(H)**, and iDISCO+ **(I)**, on E12.5 or E13.5 embryos and determined by PACA-Light. Optical images show samples after clearing. Two discs from two distinct embryo regions [E1: head (square, red), E2: body (diamond, violet)] were generated and analyzed for each sample. Each color line and bar point to assessment values of empty (black) and refractive index matching solution (blue) of each protocol. The results represent three replicates of each experiment, and data are presented as the mean ± SD (standard deviation).

In addition to rodent brains, we also compared tissues from mouse embryos at E12.5 and E13.5 cleared with the mPACT-A protocol and organic solvent-based hydrophobic tissue clearing protocols, as well as the hydrophilic tissue clearing protocols (Figure 7, Supplementary Table S2). Tissues from E2 (embryo body) showed the lowest levels of light transmittance across all protocols, while E1 (embryo head) showed the highest levels of transmittance when assessed with PACA-Light.

### 3.5 Higher Cell and Blood Vessel Density in Cerebellar Tissue May Explain the Lower Levels of Tissue Transparency

The consistent regional differences we observed in cleared brain samples, regardless of protocol, were unexpected. We therefore investigated the potential reasons for the low levels of transparency achieved in the cerebellum (B3) compared to the prefrontal cortex (B1) or the cerebral cortex (B2) at a molecular level. We performed immunostaining for lectin, glial fibrillary acidic protein (GFAP), and neurofilaments (NFs) to visualize blood vessel and neuronal networks in samples cleared with psPACT, SWITCH-4, and CUBIC-L/R. Compared to the prefrontal cortex or the cerebral cortex, the cerebellum demonstrated significantly higher cell density, in conjunction with more intricate blood vessel and neuronal networks (Supplementary Figure S15). Using a conventional confocal laser microscope, we performed 3D and volumetric imaging of blood vessel patterns after immunostaining for lectin and found that, at half depths, rat brain samples processed with psPACT and SWITCH-4 showed similar levels of transparency. We also found that, consistent with the rest of our data, the optical transparency achieved by either of the clearing protocols showed regional differences, regardless of depth (Supplementary Figure S16). These results suggest that lipid-rich and cell-dense tissues will have a lower clearing efficiency that will reduce optical transparency and antibody diffusion in the cleared brain tissue. A systematic investigation of tissue substructure effects on tissue clearing efficacy is beyond the scope of this current study, but these observations point to potential sources of the regional differences in tissue clearing observed in our study.

## 4 Discussion

The vast majority of published studies on tissue clearing have focused on developing novel clearing protocols or optimizing existing methods. Earlier studies that have provided quantitative comparisons of clearing efficacies have used diaphanometers or refractometers, but these do not provide precise measurements of transparency, nor are they readily accessible in most laboratories (Yu et al., 2021). Previous studies have measured the light transmittance of cleared samples using standard commercial spectrophotometers and provided some quantification (d'Esposito et al., 2015; Wan et al., 2018). However, some measurements of light transmittance should take into consideration aspects such as size/thickness and regional tissue characteristics.

The lack of a standardized method for generating precise, accurate quantifications of tissue transparency has created difficulties in obtaining true comparisons of the efficacies of tissue clearing protocols and pinpointing the aspects that must be further optimized. Here, we addressed these concerns by demonstrating the use of our novel PACA-Light and PACA-Glow assays to quantify tissue clearing in a systematic, high-throughput manner. Using these platforms, we were able to compare the clearing efficacies of more than 28 tissue clearing protocols in rodent brain samples, making this the largest comparative study to date. Both PACA-Light and PACA-Glow can be easily performed with standard laboratory equipment, and while the values of light transmittance obtained by the two assays are not interchangeable, we observed consistent trends between the two assays with regard to protocol efficacy.

Some published studies using cleared tissues assume that the tissues are homogeneous in their transparency, without taking into account the potential regional differences in tissue clearing. Many tissue clearing methods have been developed for targeting multiple (thus non-homogenous) tissues in the whole body. These differences are not always simply artifactual, but instead may be grounded in differences at the cellular and molecular levels that are inherent to the regions of interest. A need exists to optimize tissue clearing protocols in a region-specific manner, and failure to do so may cause individuals to miss out on key structural information about one particular region relative to another. The use of punched discs in the PACA-Light and PACA-Glow allows for the quantification of multiple tissue regions from each cleared sample. In this study, we analyzed three distinct regions of the brain: the prefrontal cortex and basal ganglia (B1), cerebral cortex and midbrain/diencephalon (B2), and cerebellum (B3). We found clear regional differences in tissue transparency that remained consistent across all tested protocols, irrespective of tissue thickness (Table 1). Specifically, cerebellar tissue consistently achieved lower levels of clearing compared to the prefrontal or cerebral cortex, regardless of the choice of tissue clearing protocol (Murakami et al., 2018).

We attribute these differences in transparency to greater cell density and the complexity of the neural and blood vessel networks observed in the cerebellum compared to the prefrontal and cerebral cortex, as assessed with GFAP, NF, lectin staining (Miyawaki et al., 2020). These results also support previous findings of a high cell density in the cerebellum and suggest that these cells may become difficult to distinguish (Keller et al., 2018). Fibrotic tissues, including the hippocampus and cerebellum, are lipid-rich regions that are difficult to clear with various tissue clearing techniques, and they show decreasing transparency (Baek et al., 2019). CLARITY-based clearing and CUBIC-L/R methods gave higher transparency levels, but we confirmed that the long duration of the denaturing detergent treatment resulted in losses of antigenicity and quenched the endogenous fluorescence at various stages of the tissue clearing process (Lee et al., 2021). We also performed 3D and volumetric imaging of blood vessels in transparent rodent brain samples and found regional differences in the optical transparency achieved by the gelation-based clearing protocols. However, due to objective lens limitations, we were unable to perform deep volumetric imaging (>5 mm). This suggests that future studies attempting to investigate three-dimensional cerebellar structure using tissue clearing methods might benefit from further optimization of various tissue clearing protocols to maximize the resolution of the structural information that can be obtained (Lagerweij et al., 2017).

These results support the use of our PACA platforms to obtain precise, reliable quantification of tissue transparency after clearing. The use of a 96-well plate makes both assays easily scalable, allowing for simultaneous analysis of up to 96 samples. This also minimizes time and resources, as well as error in data collection that occurs when analyzing samples either individually or in small groups. Supplies for PACA-Light and PACA-Glow can get easily with low cost, and it is useful with more freely available than other special equipment. Microplate spectrometer based PACA-Light is most usefulness assay in user in general laboratory. The revelation of regional differences in tissue transparency also indicates the limitations of current tissue clearing technologies. This, in turn, fuels the need for future studies aimed at improving the clearing of tissues that are hard to parse and that arguably hold greater amounts of biological information due to increased cell and/or extracellular matrix density (Ueda et al., 2020). Supplies for PACA-Light and PACA-Glow can get easily with low cost, and it is useful with more freely available than other special equipment. Microplate spectrometer based PACA-Light is most usefulness assay in user in general laboratory. The revelation of regional differences in tissue transparency also indicates the limitations of current tissue clearing technologies. Our PACA-Light and PACA-Glow assays fill a longstanding void in the field, and we believe these platforms will expedite and standardize future advancements in tissue clearing technology.

## Data Availability

The original contributions presented in the study are included in the article/Supplementary Material, further inquiries can be directed to the corresponding author.
