# Supplementary material for: Comparative Analyses of Clearing Efficacies of Tissue Clearing Protocols by Using a Punching Assisted Clarity Analysis
Source: Front Bioeng Biotechnol. 2022 Jan 28;9:784626. doi: 10.3389/fbioe.2021.784626 (PMC8831720; doi:10.3389/fbioe.2021.784626)
Supplement: Supplementary file 1 [file DataSheet1.PDF]

Supplementary Material

Supplementary Table 1. Comparison of tissue clearance achieved in mouse brains processed via various tissue clearing methods in this study.

Tissue clearing method	1.5-mm thickness mouse brain					
	PACA-Light					
	Transmittance (%)					
	OD 600			OD 850		
	B1	B2	B3	B1	B2	B3
psPACT	± 37	± 22	± 11	± 61	± 44	± 32
mPACT	35-84	19-80	11-73	63-92	47-91	25-90
SWITCH-1	± 76	± 72	± 38	± 87	± 80	± 43
Tissue-MAP	± 90	± 86	± 84	± 95	± 90	± 88
Clear^T	± 93	± 91	± 79	± 94	± 93	± 84
Sca*l*eA2	± 16	± 7	± 4	± 38	± 17	± 9
CUBIC-L/R	± 3	± 2	± 1.5	± 10	± 6	± 4
FOCM	85-93	81-89	74-87	92-98	90-97	84-94
RTF	± 46	± 30	± 24	± 71	± 57	± 45
SeeDB	± 8	± 9.5	± 7	± 29	± 25	± 21
60% TDE	± 38	± 21	± 2	± 69	± 47	± 3
80% TDE	± 30	± 10	± 3	± 58	± 21	± 18
BABB	± 32	± 21	± 13	± 64	± 53	± 36
1P-BABB	61-86	48-70	40-60	82-96	78-81	47-78
tB-BABB	± 46	± 45	± 37	± 70	± 65	± 59
3DISCO	± 82	± 72	± 37	± 91	± 76	± 55
iDISCO+	± 76	± 60	± 50	± 95	± 79	± 70
uDISCO	± 90	± 72	± 61	± 98	± 84	± 80
FDISCO	± 77	± 76	± 67	± 91	± 93	± 91
Ethanol-ECi	± 76	± 72	± 66	± 92	± 83	± 79
PEGASOS	± 73	± 61	± 45	± 95	± 84	± 50

Supplementary Table 2. Comparison of tissue clearance achieved in mouse embryos (E12.5 and E13.5) processed via various tissue clearing methods in this study.

Tissue clearing method	PACA-Light			
	Transmittance (%)			
	OD 600		OD 850	
	E1 (head)	E2 (body)	E1 (head)	E2 (body)
mPACT	± 74	±62	± 79	± 72
Clear^T	± 4	± 3	± 19	± 14
Sca*l*eA2	± 6	± 1	± 12	± 3
CUBIC-L/R	± 74	± 49	± 92	± 80
FOCM	± 20	± 6	± 38	± 24
SeeDB	± 20	± 20	± 40	± 39
BABB	± 77	± 60	± 82	± 77
iDISCO+	± 59	± 57	± 81	± 85

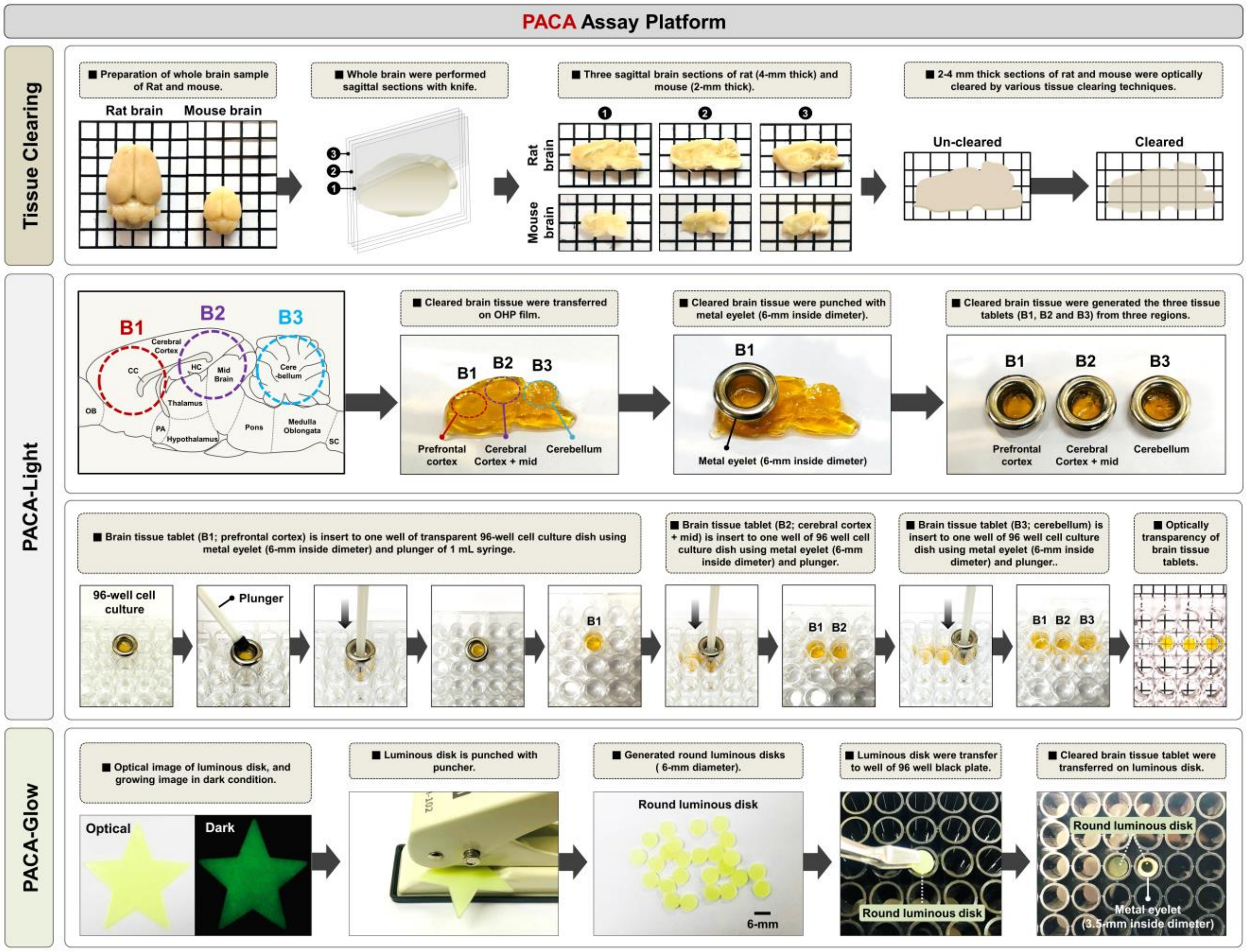

Supplementary Figure 1. Schematic representation of PACA-Light and PACA-Glow.

1) Cleared sample preparation for measuring tissue transparency. After generation of transparent brains in various tissue clearing methods, cleared brain samples were punched using metal eyelets either 3.5 or 6-mm in diameter. **2)** PACA-Light: Each tablet was transferred to an individual well of a clear 96-well plate. **3)** PACA-Glow: Each luminous disk was transferred to an individual well of a black, opaque 96-well plate.

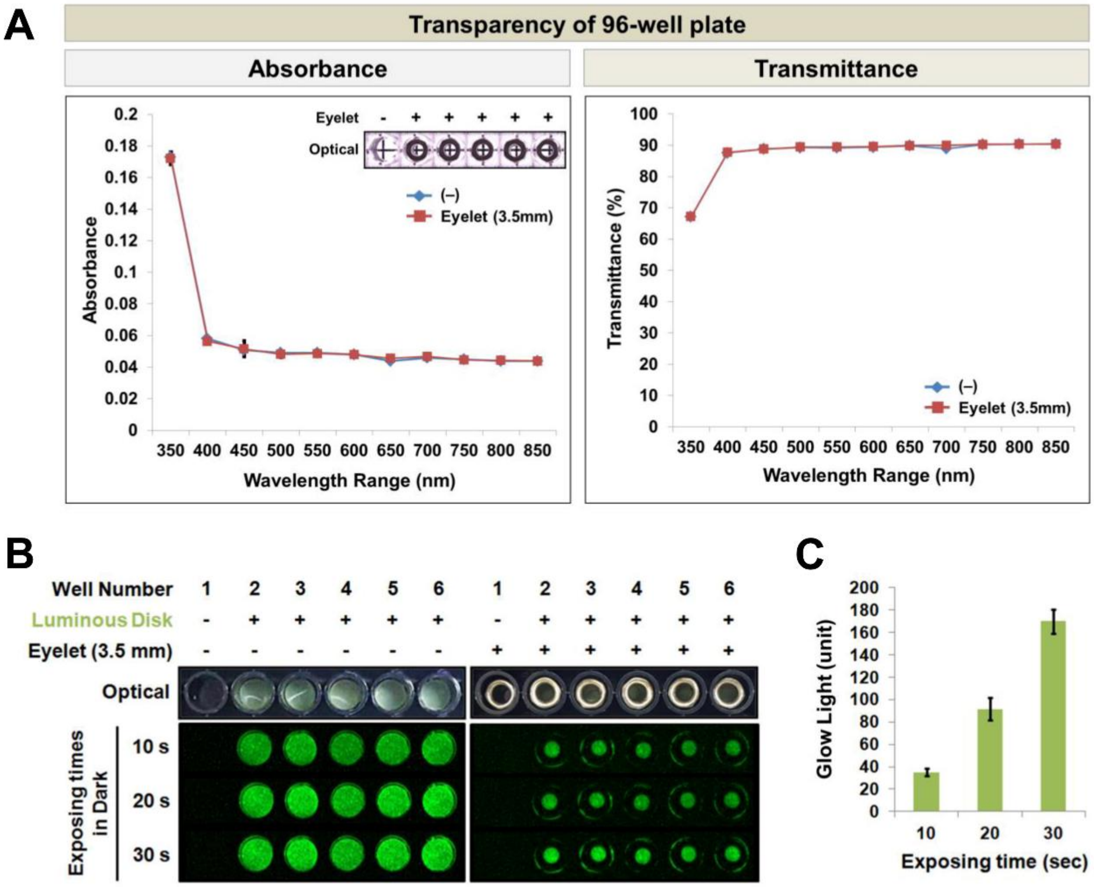

Supplementary Figure 2. Quantification of light transmitted of the PACA platform.

Absorbance and transmittance (%) of transparent 96-well plate (empty; diamond, blue) **(A)** including metal eyelets (square, red) either 3.5mm in diameter at 350-850 nm. **(B)** Comparison of light emitted by the luminous disk including metal eyelet for 10-30 sec in dark. **(C)** Quantifying the amount of light transmitted from luminous disk through the 3.5-mm metal eyelet of **(B)**. Green bar point to assessment values of glow light (unit) in dark. Results reflect three replicates of each experiment, and data are presented as the mean ± SD (standard deviation).

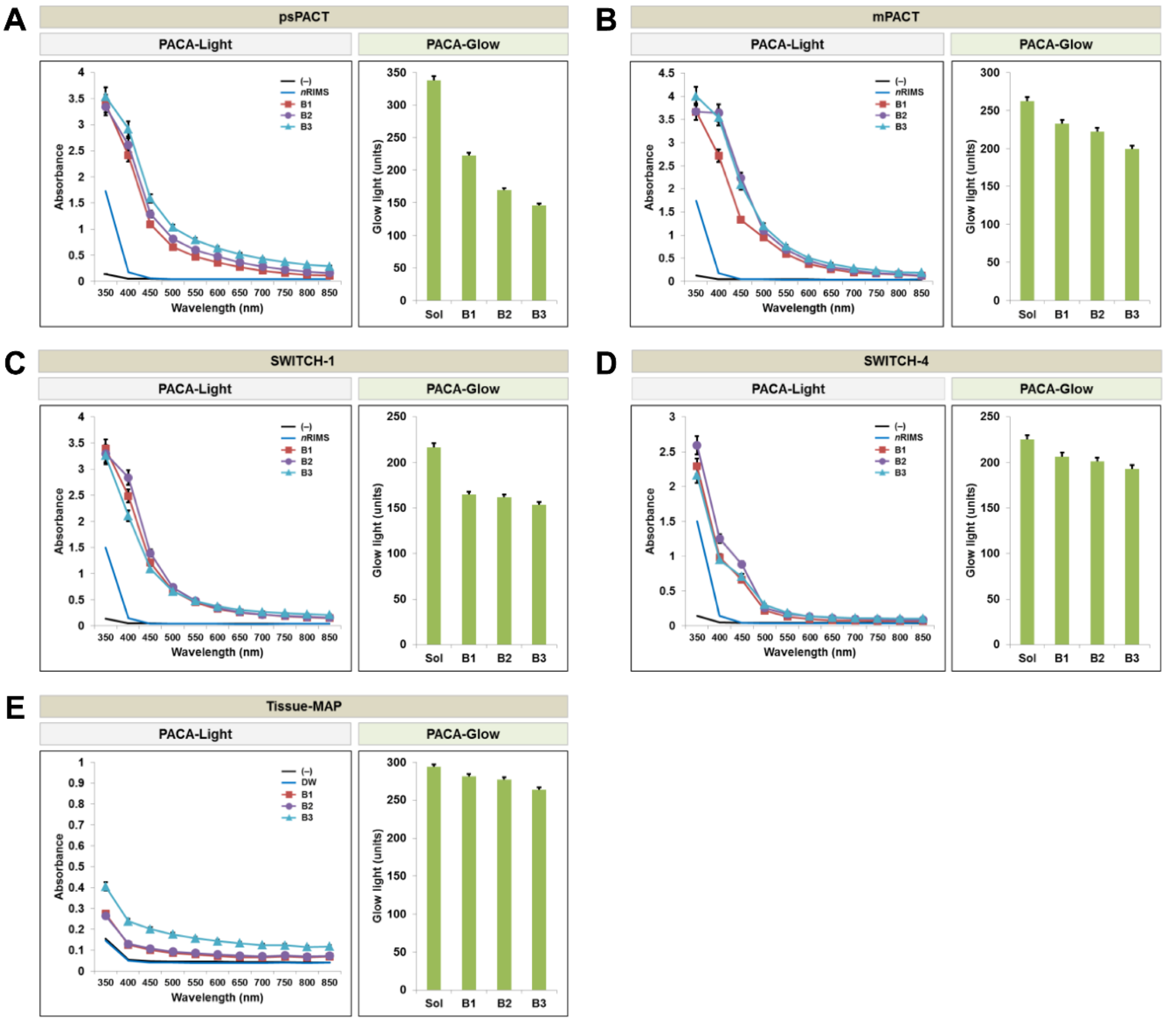

Supplementary Figure 3. Absorbance and glow light of optically cleared rodent brain via gelation-based tissue clearing protocols.

Absorbance and glow light (units) of transparent rodent brain tissue tablets of psPACT **(A)**, mPACT **(B)**, SWITCH-1 **(C)**, SWITCH-4 **(D)**, and Tissue-MAP **(E)** on rodent brain samples via both PACA-Light and PACA-Glow. Each color line point to assessment values of empty (black) and refractive index matching solution (blue) of each protocol, and three distinct regions of the brain the B1 (square, red), B2 (diamond, violet), and B3 (triangle, sky blue). Green bar point to assessment values of glow light (unit) in dark. Results reflect three replicates of each experiment, and data are presented as the mean ± SD (standard deviation).

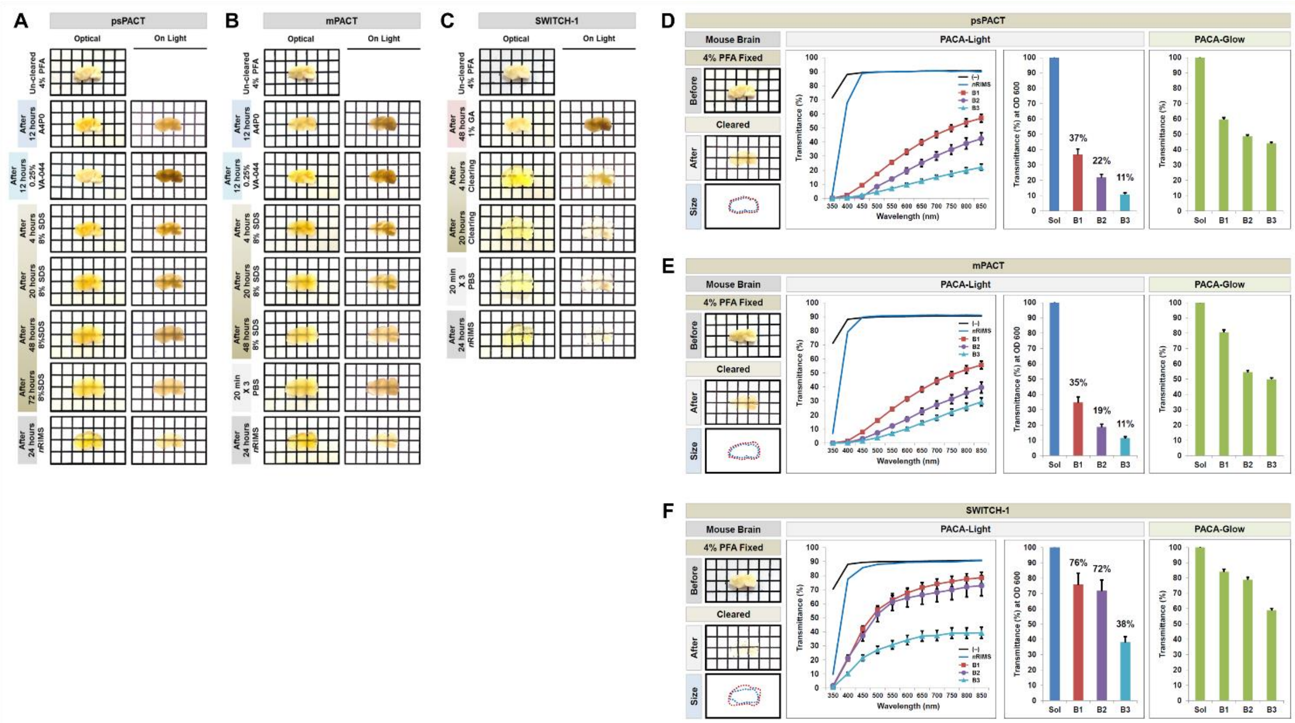

Supplementary Figure 4. Comparison of tissue clearance achieved in mouse brain via gelation-based tissue clearing protocols.

Comparison of optical clearing process of psPACTs **(A)**, mPACT **(B)**, and SWITCH-1 (C) in mouse brain samples (1.5-mm thickness). Comparison of clearing efficacies of psPACT **(D)**, mPACT (E), and SWITCH-1 **(F)** in mouse brain samples via both PACA-Light and PACA-Glow. Optical images showing samples before (blue dot line) and after clearance (red dot line), along with any changes in sample size upon tissue processing, are included (see also **Supplementary Figure 12**). Three tablets from three distinct brain regions (B1: prefrontal cortex and basal ganglia, B2: cerebral cortex and midbrain/diencephalon, B3: cerebellum) were generated and analyzed for each sample. Each color line and bar point to assessment values of empty (black) and refractive index matching solution (blue) of each protocol, and three distinct regions of the brain the B1 (square, red), B2 (diamond, violet), and B3 (triangle, sky blue). Green bar point to assessment values of glow light (unit) in dark. Results reflect three replicates of each experiment, and data are presented as the mean ± SD (standard deviation). The transparency of the cleared brain was evident against a patterned background (length:width=5 mm:5 mm).

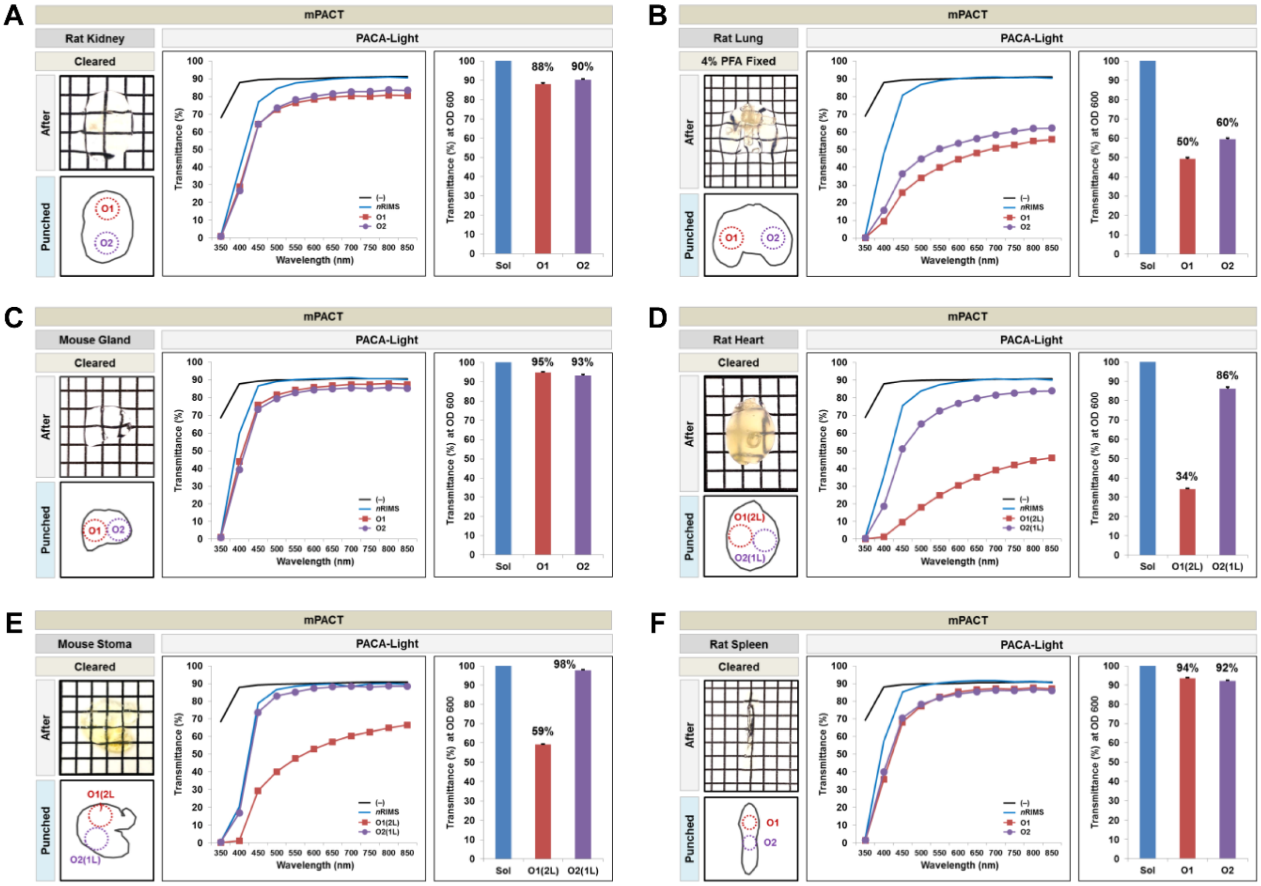

Supplementary Figure 5. Comparison of non-CNS tissue clearance achieved via mPACT protocols.

Comparison of mPACT clearing efficacies of kidney **(A)**, lung **(B)**, gland **(C)**, heart **(D)**, stoma **(E)**, and spleen **(F)** in rodent organs samples via both PACA-Light and PACA-Glow. Optical images showing samples after clearance, along with punched regions in sample, are included. L1 (red) and L2 (violet) indicate that membrane layers of organs, such as heart and stoma. Two tablets from two distinct organ regions (O1 or O1(2L) (red), O2 or O2(1L) (violet)) were generated and analyzed for each sample. Each color line and bar point to assessment values of empty (black) and refractive index matching solution (*n*RIMS, blue). Results reflect three replicates of each experiment, and data are presented as the mean ± SD (standard deviation).

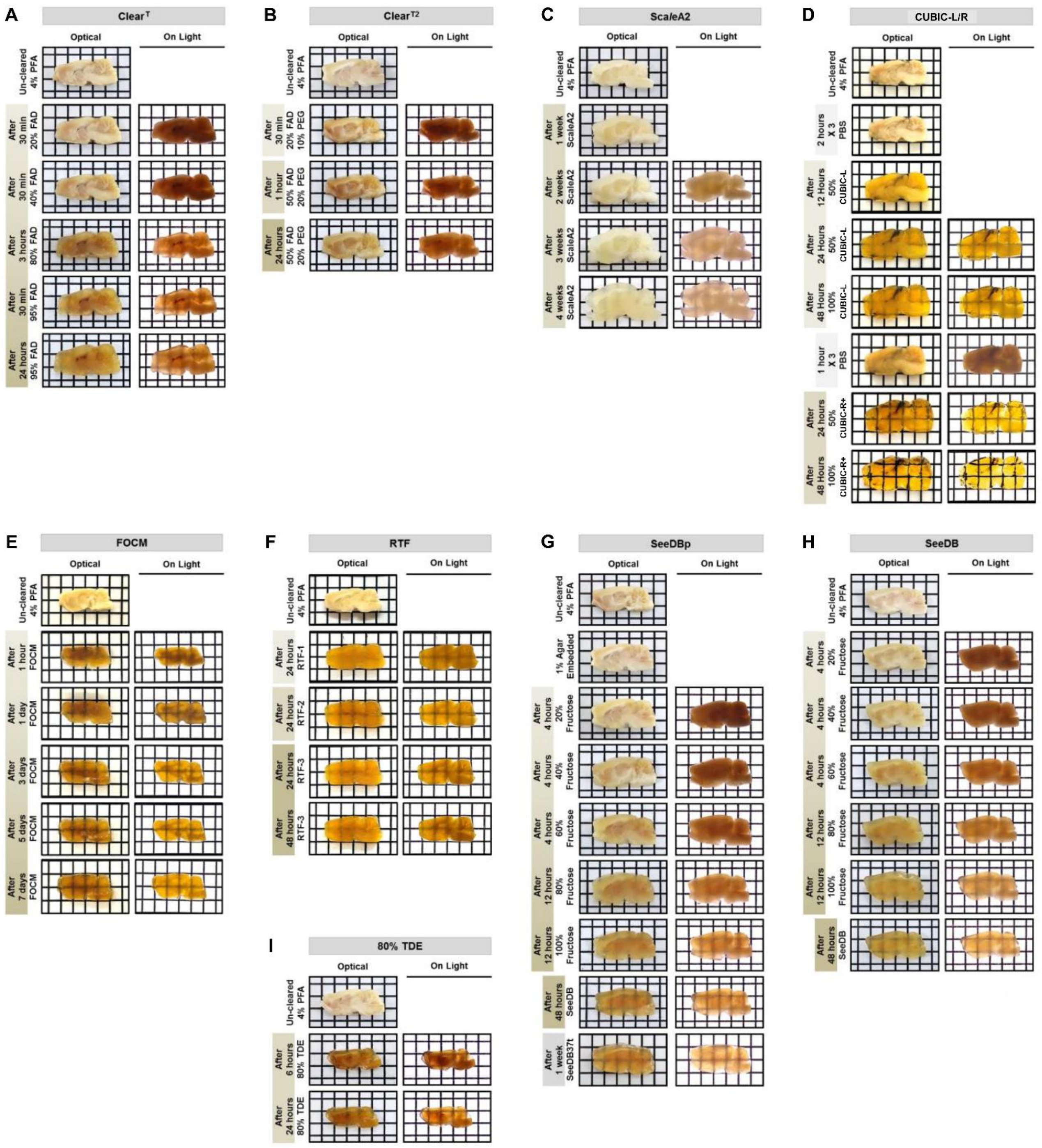

Supplementary Figure 6. Comparison of tissue clearance process achieved via nine hydrophilic clearing methods in rat brain.

Comparison of optical clearing process of *Clear^{T}* **(A)**, *Clear^{T2}* **(B)**, Sca*l*eA2 **(C)**, CUBIC-L/R **(D)**, FOCM **(E)**, RTF **(F)**, SeeDB **(G)**, SeeDBp **(H)**, and 80% TDE **(I)** on rat brain samples (3-mm thickness). Optical images showing samples before and after clearance, along with any changes in sample size upon tissue processing, are included. The size of all brain thick was compared with un-

cleared (blue dot line) and final cleared (red dot line). The transparency of the cleared brain was evident against a patterned background (length:width=5 mm:5 mm).

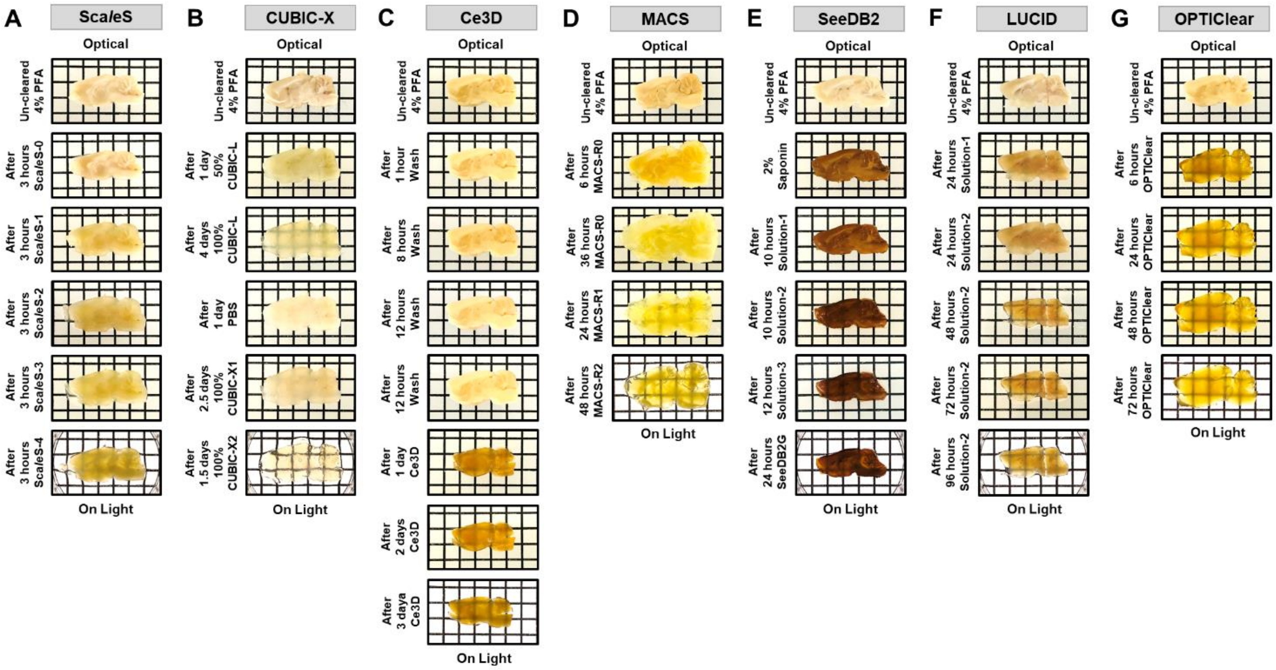

Supplementary Figure 7. Comparison of tissue clearance process achieved via seven hydrophilic clearing methods in rat brain.

Comparison of optical clearing process of Sca*le*S **(A)**, CUBIC-X **(B)**, Ce3D **(C)**, MACS **(D)**, SeeDB2 **(E)**, LUCID **(F)**, and OPTIClear **(G)** on rat brain samples (3-mm thickness). Optical images showing samples before and after clearance, along with any changes in sample size upon tissue processing, are included. The transparency of the cleared brain was evident against a patterned background (length:width=5 mm:5 mm).

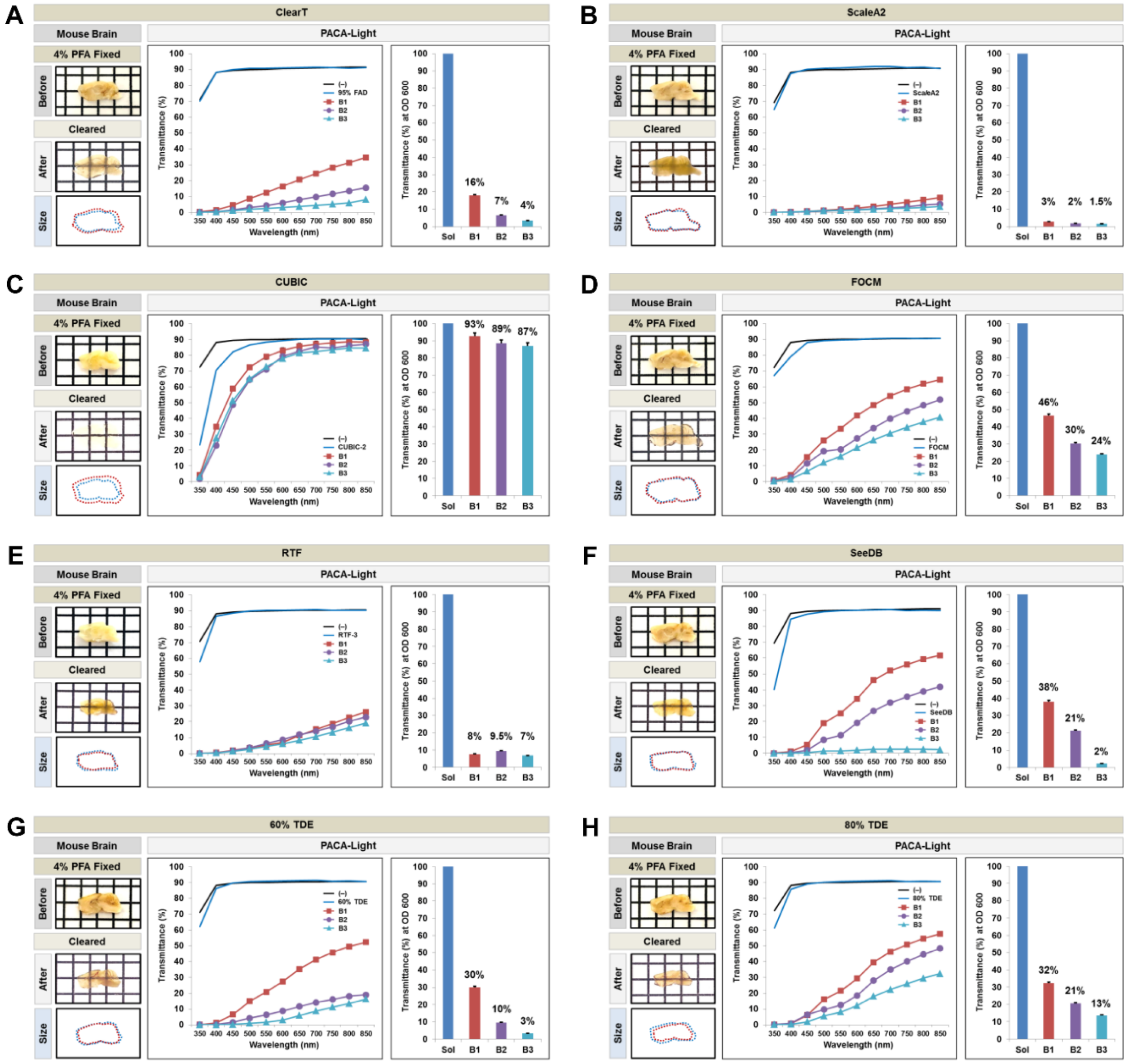

Supplementary Figure 8. Comparison of tissue clearance achieved via clearing methods based on hyperhydration or aqueous solutions with high-refractive indices in mouse brain.

Comparison of clearing efficacies of *Clear*^{T} **(A)**, Sca*l*eA2 **(B)**, CUBIC-L/R **(C)**, FOCM **(D)**, RTF **(E)**, SeeDB **(F)**, 60% TDE **(G)**, and 80% TDE **(H)** on mouse brain samples (1.5-mm thickness) via PACA-Light. Optical images showing samples before and after clearance, along with any changes in sample size upon tissue processing, are included. Three tablets from three distinct brain regions (B1: prefrontal cortex and basal ganglia, B2: cerebral cortex and midbrain/diencephalon, B3: cerebellum) were generated and analyzed for each sample. Each color line and bar point to assessment values of empty (black) and refractive index matching solution (blue) of each protocol, and three distinct regions of the brain the B1 (square, red), B2 (diamond, violet), and B3 (triangle, sky blue). Results reflect three replicates of each experiment, and data are presented as the mean ± SD (standard deviation).

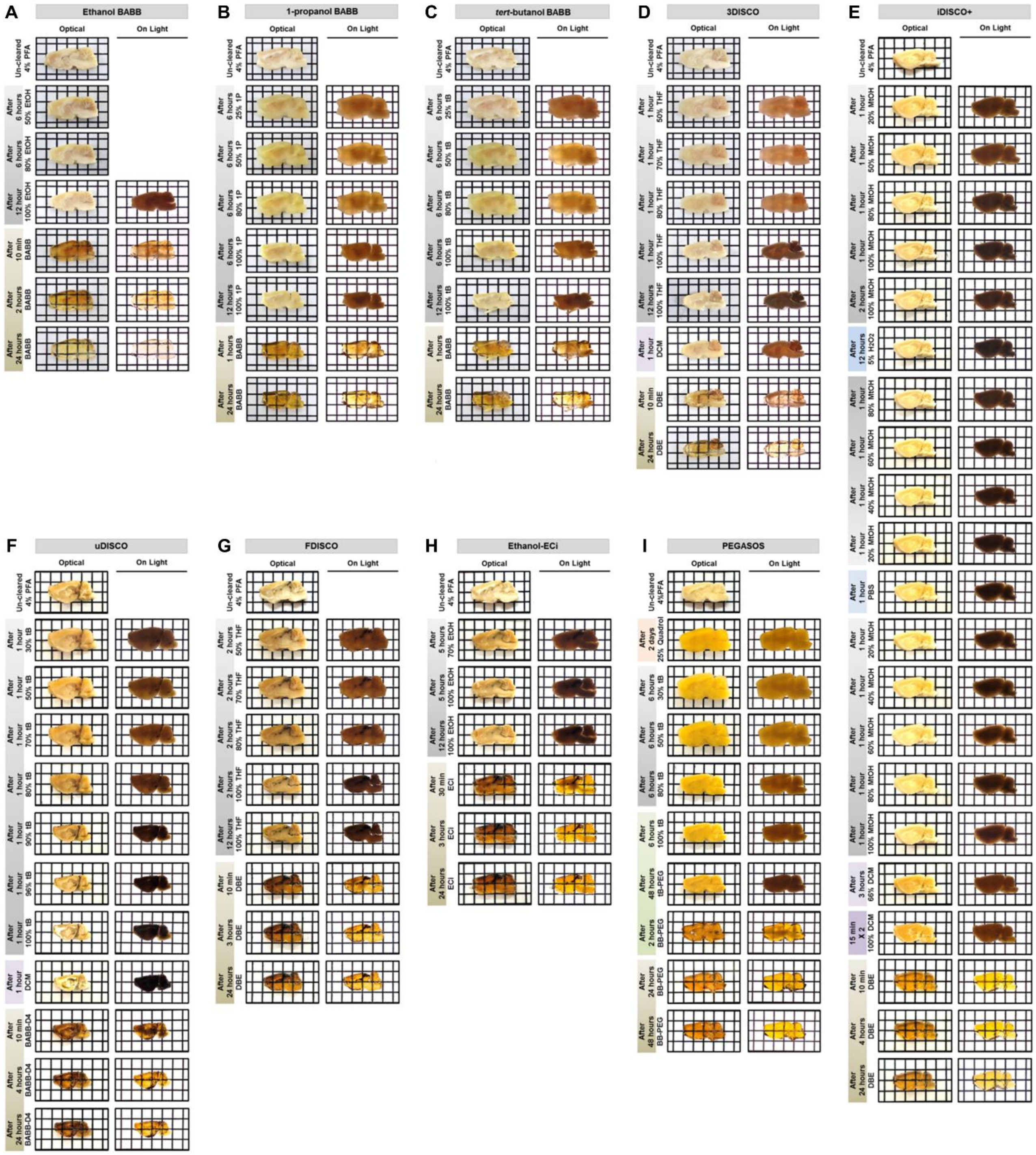

Supplementary Figure 9. Comparison of tissue clearance process achieved in rat brains processed via organic solvent-based clearing protocols.

Comparison of optical clearing process of Ethanol BABB **(A)**, 1-propanol BABB **(B)**, *tert*-butanol BABB **(C)**, 3DISCO **(D)**, iDISCO+ **(E)**, uDISCO **(F)**, FDISCO **(G)**, Ethanol-ECi **(H)**, and PEGASOS **(i)** on rat brain samples (3-mm thickness). Optical images showing samples before and after clearance, along with any changes in sample size upon tissue processing, are included. The size of all brain thick

was compared with un-cleared (blue dot line) and final cleared (red dot line). The transparency of the cleared brain was evident against a patterned background (length:width=5 mm:5 mm).

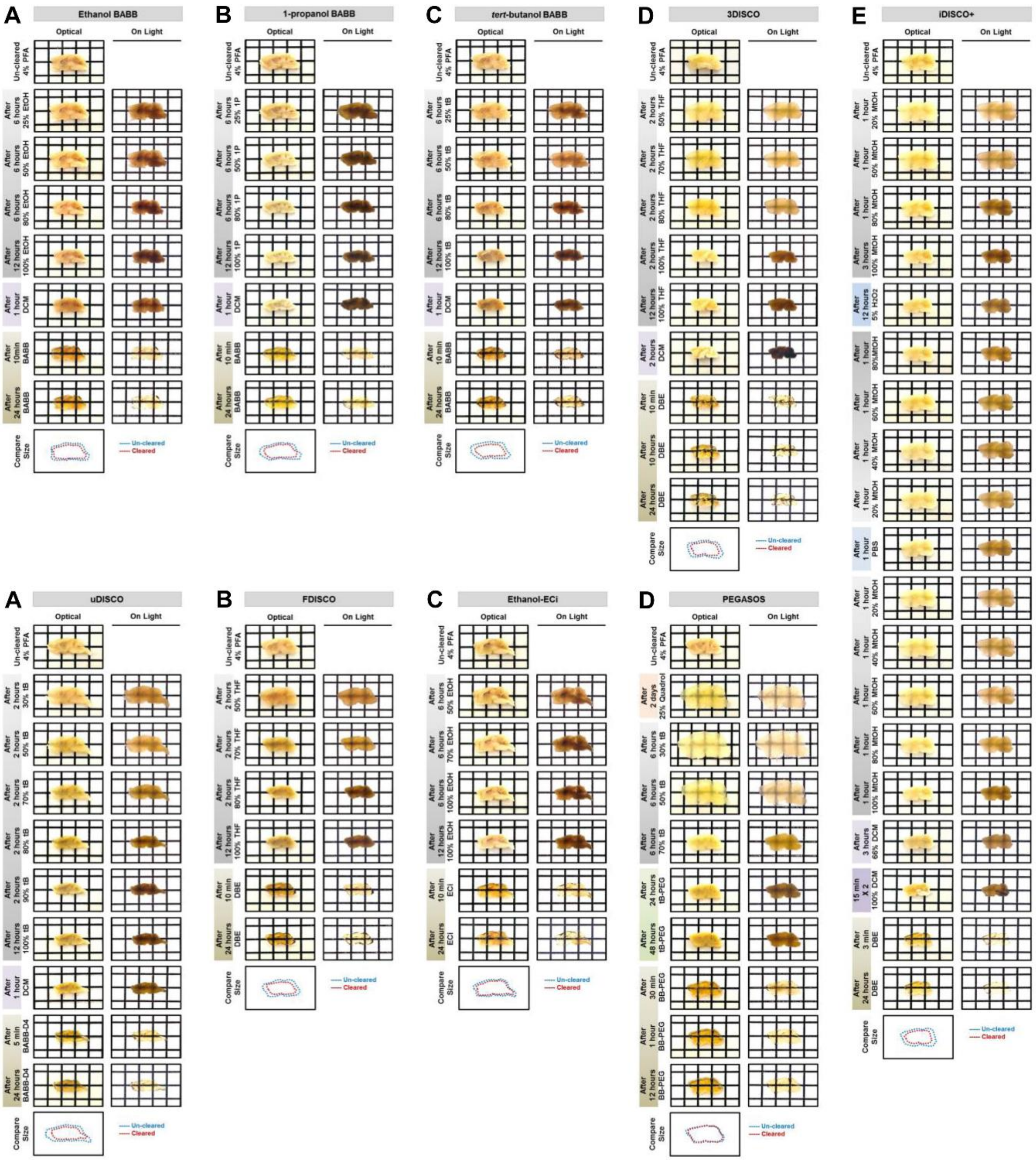

Supplementary Figure 10. Comparison of tissue clearance process achieved in mouse brains processed via organic solvent-based clearing protocols.

Comparison of clearing efficacies of Ethanol BABB **(A)**, 1-propanol BABB **(B)**, *tert*-butanol BABB **(C)**, 3DISCO **(D)**, iDISCO+ **(E)**, uDISCO **(F)**, FDISCO **(G)**, Ethanol-ECi **(H)**, and PEGASOS **(I)** on mouse brain samples (1.5-mm thickness). Optical images showing samples before and after clearance, along with any changes in sample size upon tissue processing, are included. The size of all brain thick

was compared with un-cleared (blue dot line) and final cleared (red dot line). The transparency of the cleared brain was evident against a patterned background (length:width=5 mm:5 mm).

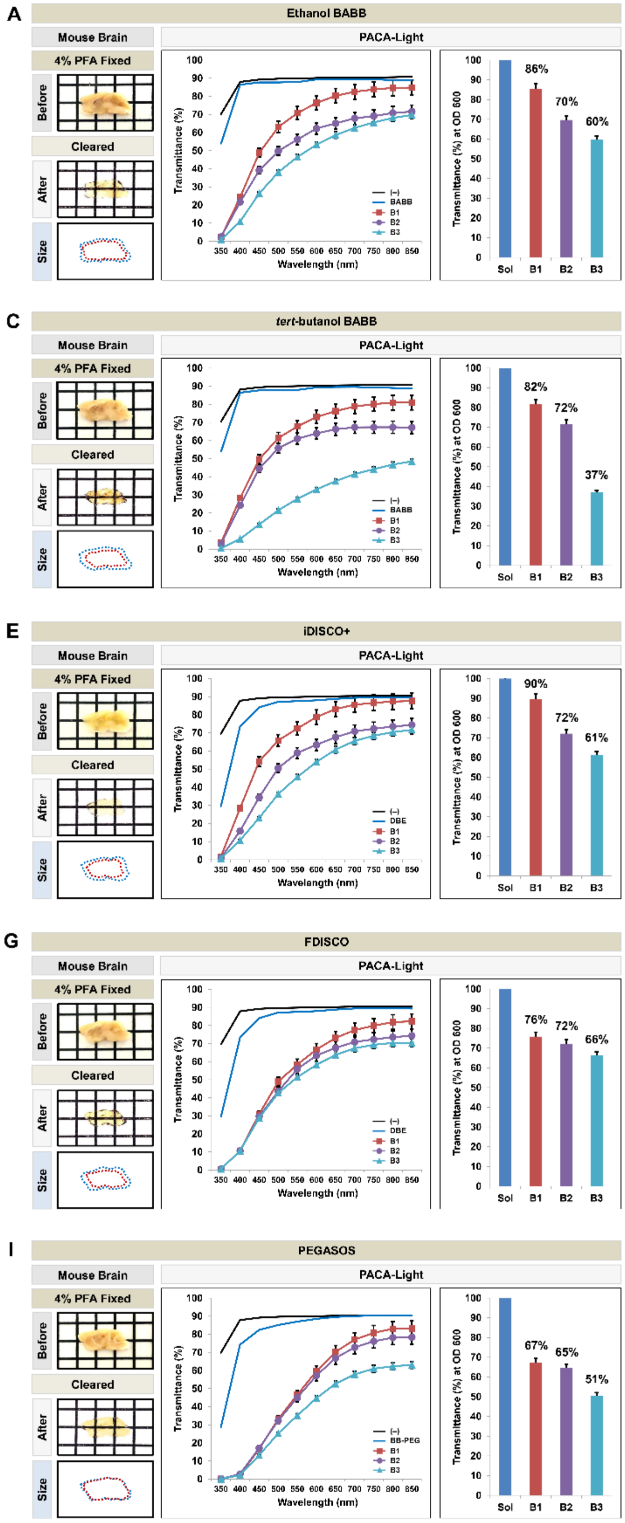

Supplementary Figure 11. Comparison of tissue clearance achieved in mouse brains processed via organic solvent-based clearing protocols.

Comparison of clearing efficacies of Ethanol BABB **(A)**, 1-propanol BABB **(B)**, *tert*-butanol BABB **(C)**, 3DISCO **(D)**, iDISCO+ **(E)**, uDISCO **(F)**, FDISCO **(G)**, Ethanol-ECi **(H)**, and PEGASOS **(I)** on

mouse brain samples (1.5-mm thickness) via PACA-Light. Optical images showing samples before (blue dot line) and after clearance (red dot line), along with any changes in sample size upon tissue processing, are included (see also **Supplementary Figure 12**). Three tablets from three distinct brain regions (B1: prefrontal cortex and basal ganglia, B2: cerebral cortex and midbrain/diencephalon, B3: cerebellum) were generated and analyzed for each sample. Each color line and bar point to assessment values of empty (black) and refractive index matching solution (blue) of each protocol, and three distinct regions of the brain the B1 (square, red), B2 (diamond, violet), and B3 (triangle, sky blue). Results reflect three replicates of each experiment, and data are presented as the mean ± SD (standard deviation). The transparency of the cleared brain was evident against a patterned background (length:width=5 mm:5 mm).

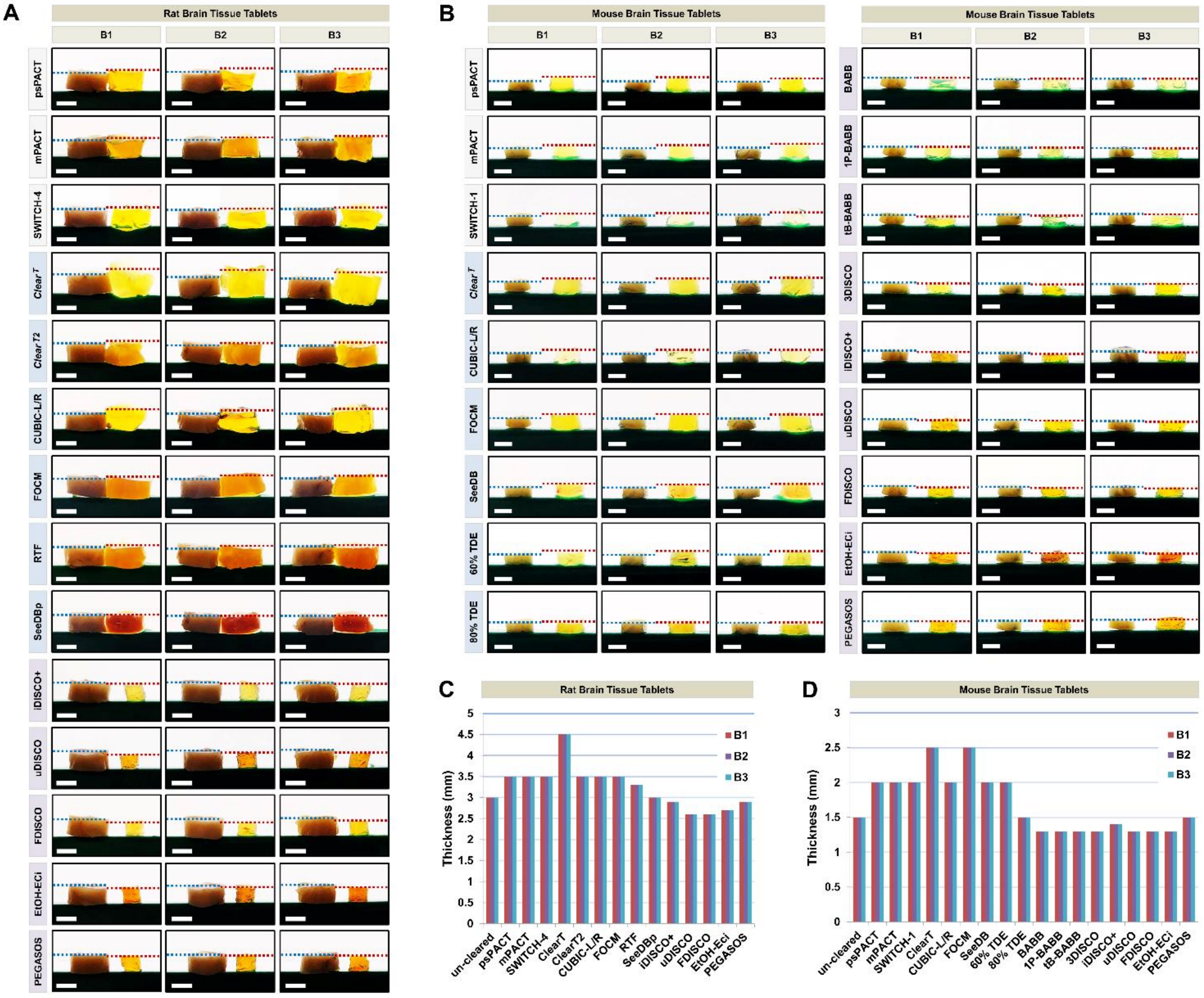

Supplementary Figure 12. Comparison of tissue thickness in rodent brain tissue tablets processed via various clearance protocols.

(A and C) Comparison of thickness of three distinct rat brain tissue tablets (3-mm thickness) in before (left) and after clearance (right) via 14 tissue clearance protocols, such as psPACT, mPACT, SWITCH-4, *Clear*^{T}, *Clear*^{T2}, CUBIC, FOCM, RTF, SeeDBp, iDISCO+, uDISCO, FDISCO, Ethanol-ECi, and PEGASOS. **(B and D)** Comparison of thickness of three distinct mouse brain tissue tablets (1.5-mm thickness) in before (left) and after clearance (right) via 21 tissue clearance protocols, such as psPACT, mPACT, SWITCH-1, *Clear*^{T}, CUBIC-L/R, FOCM, SeeDB, 60% TDE, 80% TDE, BABB, 1-propanol BABB, *tert*-butanol BABB, 3DISCO, iDISCO+, uDISCO, FDISCO, Ethanol-ECi, and PEGASOS. The thickness of all brain thick was compared with un-cleared (blue dot line) and final cleared (red dot line). Three color bar indicate to three distinct regions of the brain the B1 (square, red), B2 (diamond, violet), and B3 (triangle, sky blue). Results reflect three replicates of each experiment, and data are presented as the mean ± SD (standard deviation). Scale bar (White: 3-mm).

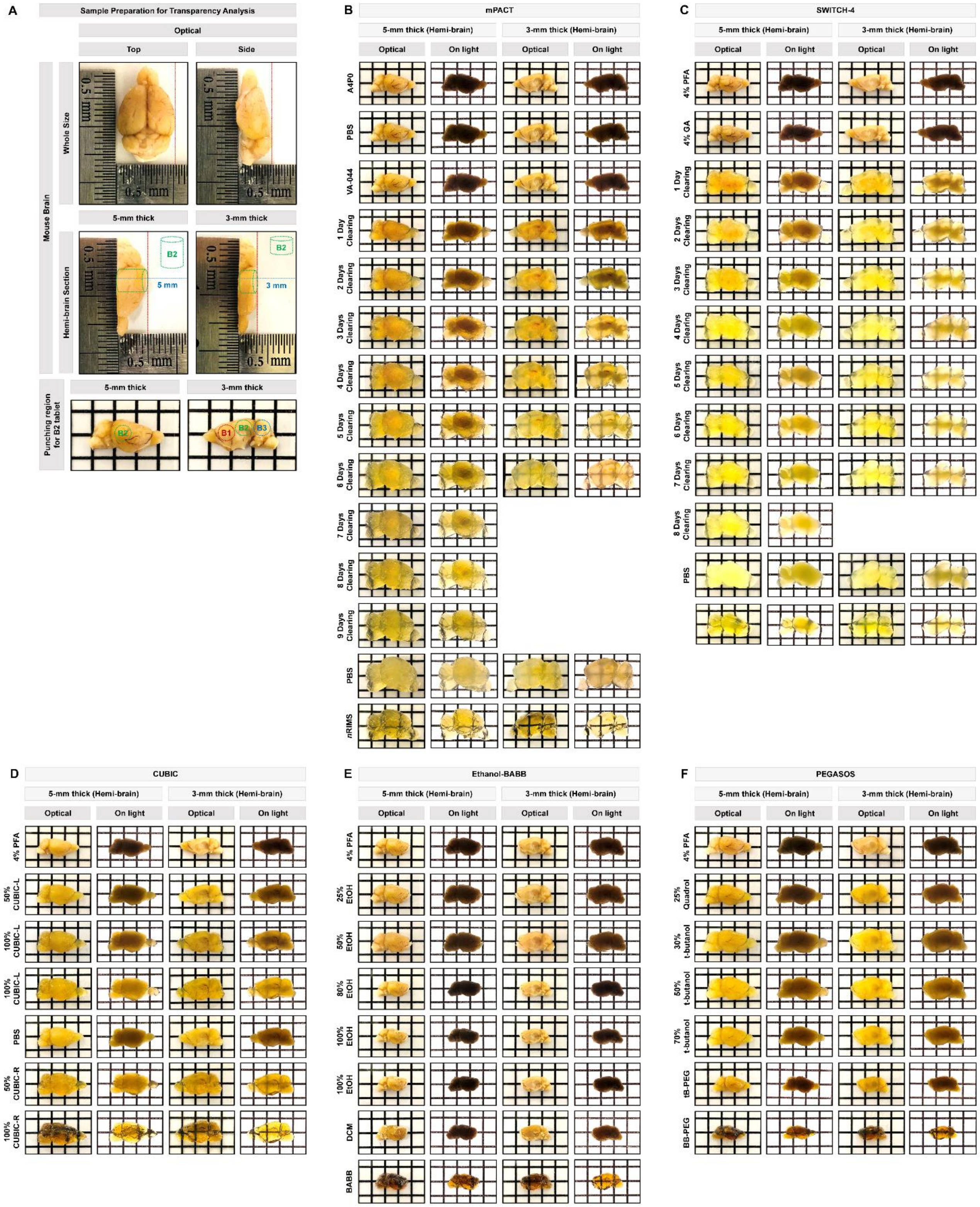

Supplementary Figure 13. Comparison of the tissue clearing process achieved in mouse hemi-brains processed with various clearing protocols.

(A) Optical images showing uncleared mouse brain sample fixed with 4% PFA. Comparison of the thickness of whole brain (width: 10 × 5 mm) and hemi-sections (3 mm and 5 mm thick). Three circles point to the areas of the discs (B1: red, B2: green, and B3: blue) used to measure transmittance with PACA-Light. Comparison of clearing efficacies of mPACT **(B)**, SWITCH-4 **(C)**, CUBIC-L/R **(D)**, Ethanol BABB **(E)**, and PEGASOS **(F)** on mouse hemi-brain samples (3 mm and 5 mm thickness). Optical images show samples before and after clearing, as well as any changes in sample size upon tissue processing. The transparency of the cleared brain was evident against a patterned background (length:width=5 mm:5 mm).

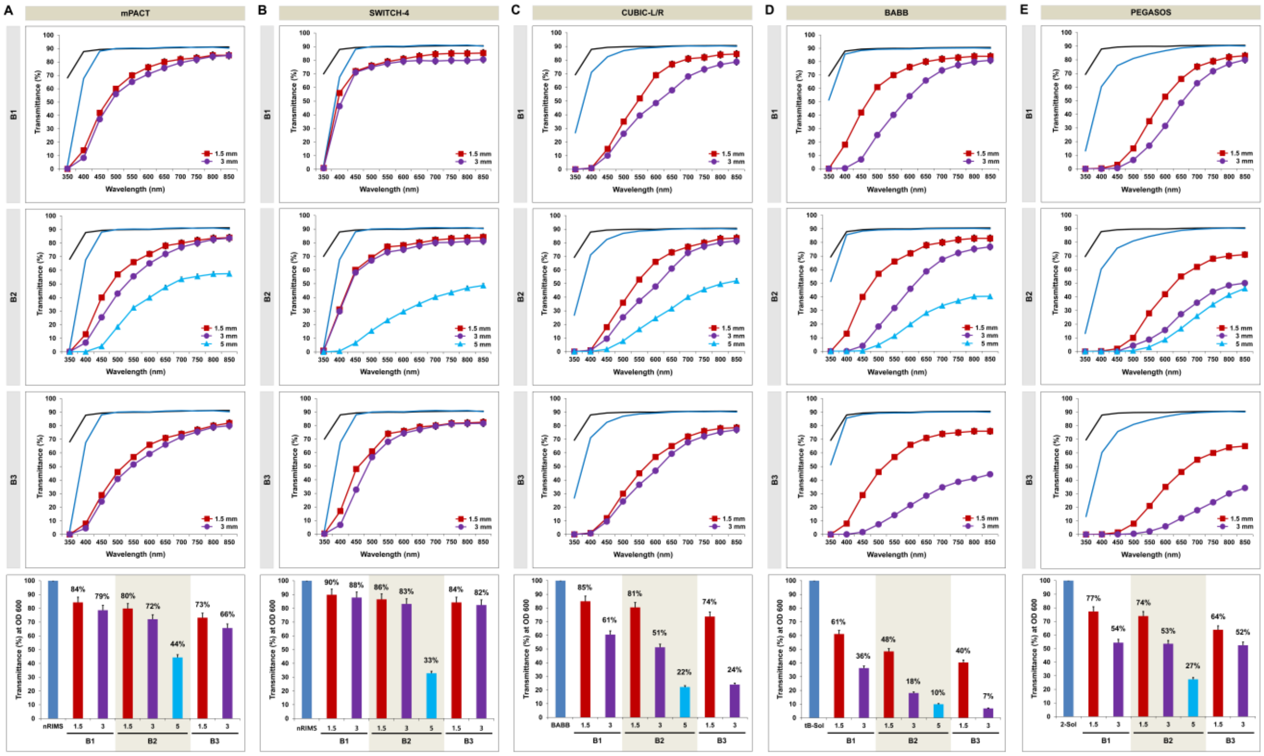

Supplementary Figure 14. Comparison of tissue clearing achieved in mouse hemi-brains of different thicknesses.

Comparison of clearing efficacies of mPACT **(A)**, SWITCH-4 **(B)**, CUBIC **(C)**, BABB **(D)**, and PEGASOS **(E)** for mouse brain samples from three distinct regions (B1, B2, and B3) and different thicknesses (1.5 mm, 3 mm, and 5 mm) with PACA-Light. Three discs from three distinct brain regions (B1: prefrontal cortex and basal ganglia, B2: cerebral cortex and midbrain/diencephalon, B3: cerebellum) were generated and analyzed for each sample. Each color line and bar point to assessment values of empty (black) and refractive index matching solution (blue) for each protocol, and three different thicknesses of mouse brain discs: 1.5 mm (square, red), 3 mm (diamond, violet), and 5 mm (triangle, sky blue). Results represent three replicates of each experiment, and data are presented as the mean ± SD (standard deviation).

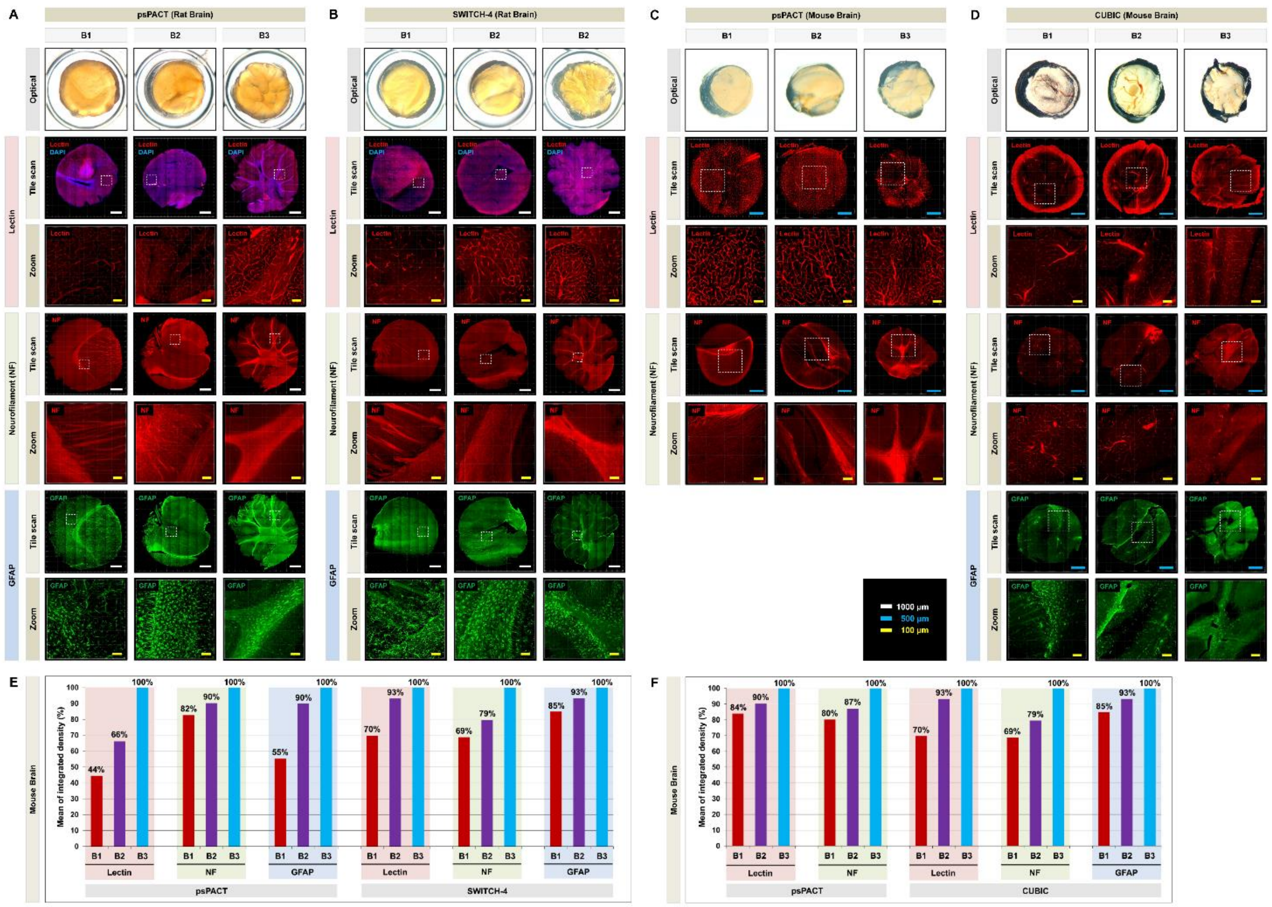

Supplementary Figure 15. Immunofluorescence investigation of regional differences in tissue transparency in rodent brains.

Discs were generated from three distinct regions (B1: prefrontal cortex and basal ganglia, B2: cerebral cortex and midbrain/diencephalon, B3: cerebellum) of rat and mouse brain samples and processed with psPACT **(A and C)**, SWITCH-4 **(B),** or CUBIC-L/R **(D)**. Immunostaining of rat and mouse brain disc tissues was performed with lectin to visualize blood vessels and with glial fibrillary acidic protein (GFAP) and neurofilament (NF) to visualize neurons. The upper images (x and y = 850 µm, z = 100 µm) were 8 × 8 (rat) or 4 × 4 (mouse) images tiled with 10× magnification. The 3D projections are shown for each image, focusing on the yellow box region; the lower images were created from single z-images (x and y = 850 µm, z = 100-200 µm). Scale bar (White: 1000 µm; Blue: 500 µm; Yellow: 100 µm). **(E and F)** Comparison of integrated density as a function of single z-images for lectin, NF, and GFAP in rat and mouse brain discs **(A-D)**. The fluorescence signals of single z-stack images (rat: 10 tiles, mouse: 4 tiles) were measured as integrated densities (ImageJ software). The mean values are shown for the green fluorescence signal of single z-stack images. Integrated density (%) was represented relative to the normalized values of maximal effects of fluorescence signals. Fluorescence intensity (cell density) of B1 and B2 was normalized to fluorescence intensity of B3 (high density; 100%). Each color line points to assessment values of three distinct regions of the brain: B1 (red), B2 (violet), and B3 (sky blue).

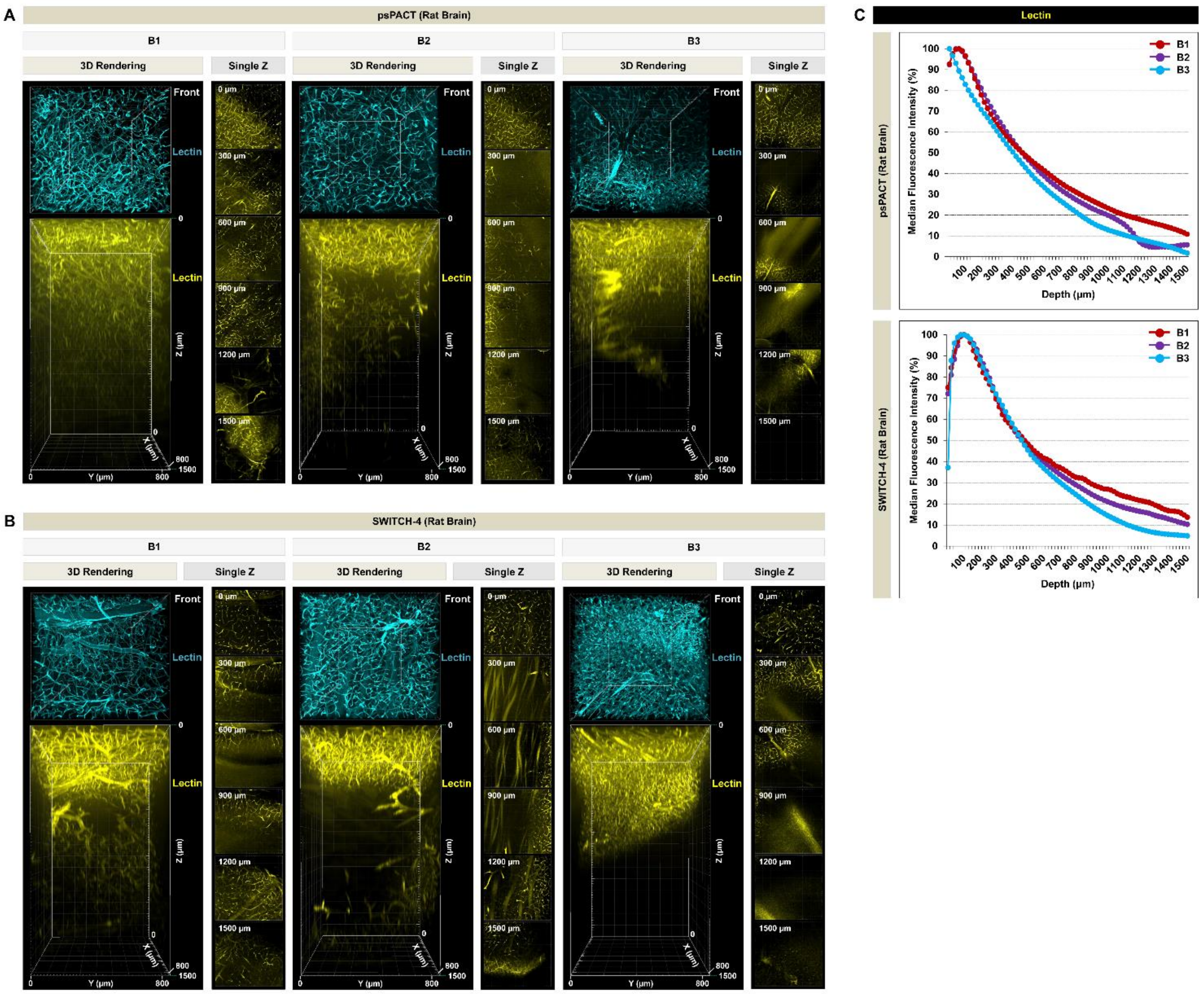

Supplementary Figure 16. 3D reconstruction of rat brain microvasculature with psPACT and SWITCH-4.

(A and B) The 3D and volumetric imaging of lectin-labeled rat brain tissue (B1: prefrontal cortex and basal ganglia, B2: cerebral cortex and midbrain/diencephalon, B3: cerebellum), cleared with the psPACT or SWITCH-4 procedures. 3D reconstruction of 1500 pictures, starting at the parietal cortical surface and moving to 1500 µm below. Images created from serial single z-images (x and y = 850 µm, z = 1-1500 µm) of the blood vessel pattern using confocal microscopy in three distinct regions. **(C)** Relative fluorescence intensity as a function of imaging depth (1-1500 µm) and volumetric imaging of psPACT and SWITCH-4 rat brain microvasculature. Each color line points to assessment values of three distinct regions of the brain: B1 (red), B2 (violet), and B3 (sky blue).
